# Oxylipins are implicated as communication signals in tomato–root-knot nematode (*Meloidogyne javanica*) interaction

**DOI:** 10.1038/s41598-020-79432-6

**Published:** 2021-01-11

**Authors:** Nathalia Fitoussi, Eli Borrego, Michael V. Kolomiets, Xue Qing, Patricia Bucki, Noa Sela, Eduard Belausov, Sigal Braun Miyara

**Affiliations:** 1 Department of Entomology, Nematology and Chemistry Units, Agricultural Research Organization (ARO), The Volcani Center, P.O. Box 15159, 50250 Rishon LeZion, Bet Dagan, Israel; 2 Department of Plant Pathology and Microbiology, The Faculty of Agriculture, Food and Environment, The Hebrew University of Jerusalem, 76100 Rehovot, Israel; 3 Thomas H. Gosnell School of Life Sciences, Rochester Institute of Technology, Rochester, NY 14623 USA; 4 Department of Plant Pathology and Microbiology, Texas A&M University, TAMU 2132, College Station, 77843-2132 USA; 5 Department of Plant Protection, Nanjing Agricultural University, Nanjing, China; 6 Department of Plant Pathology and Weed Research, ARO, The Volcani Center, 50250 Bet Dagan, Israel; 7 Department of Plant Sciences, Ornamental Plants and Agricultural Biotechnology, ARO, The Volcani Center, 50250 Bet Dagan, Israel

**Keywords:** Effectors in plant pathology, Jasmonic acid, Biotic, Plant stress responses

## Abstract

Throughout infection, plant-parasitic nematodes activate a complex host defense response that will regulate their development and aggressiveness. Oxylipins—lipophilic signaling molecules—are part of this complex, performing a fundamental role in regulating plant development and immunity. At the same time, the sedentary root-knot nematode *Meloidogyne* spp. secretes numerous effectors that play key roles during invasion and migration, supporting construction and maintenance of nematodes' feeding sites. Herein, comprehensive oxylipin profiling of tomato roots, performed using LC–MS/MS, indicated strong and early responses of many oxylipins following root-knot nematode infection. To identify genes that might respond to the lipidomic defense pathway mediated through oxylipins, RNA-Seq was performed by exposing *Meloidogyne javanica* second-stage juveniles to tomato protoplasts and the oxylipin 9-HOT, one of the early-induced oxylipins in tomato roots upon nematode infection. A total of 7512 differentially expressed genes were identified. To target putative effectors, we sought differentially expressed genes carrying a predicted secretion signal peptide. Among these, several were homologous with known effectors in other nematode species; other unknown, potentially secreted proteins may have a role as root-knot nematode effectors that are induced by plant lipid signals. These include effectors associated with distortion of the plant immune response or manipulating signal transduction mediated by lipid signals. Other effectors are implicated in cell wall degradation or ROS detoxification at the plant–nematode interface. Being an integral part of the plant's defense response, oxylipins might be placed as important signaling molecules underlying nematode parasitism.

## Introduction

*Meloidogyne* species of root-knot nematodes (RKN) are one of the main devastating plant parasites, infecting an estimated 5000 plant species^1–3^. The RKN are obligatory sedentary endoparasitic biotrophs, with more than 90 known *Meloidogyne* species distributed ubiquitously. The most destructive species are *M. incognita, M. arenaria, M. hapla* and *M. javanica*, causing crop losses amounting to hundreds of billions of US dollars each year^4–6^. Successful parasite penetration, migration, establishment and maintenance rely mainly on the secretion of effectors through the stylet that promote and establish an intimate long-term interaction with the host^7–9^. These effectors are predominantly synthesized by the esophageal glands, two subventral and one dorsal; and other organs, such as amphids and cuticle, also participate in the parasitic secretion^10^. Multiple effectors of plant-parasitic nematode species have been successfully isolated and characterized using transcriptome, secretome and RNAi approaches^11–19^. Of the various effector categories, some effectors have been found to act as immunomodulators that manipulate, via mimicry or suppression, the host immune system.

Although lipids are vital cellular components, increasing evidence suggests their supplementary role in plant immunity. Lipids and their derivative signaling molecules are involved in responses to biotic and abiotic stresses^20,21^. Several studies have suggested that these compounds are subject to pathogen manipulation and interference. Nematode-derived fatty acid- and retinol-binding (FAR) proteins are predominantly secreted into the host cell for developmental processes, but may facilitate parasitism by interfering with lipid signaling related to plant defense^22–24^. Similarly, in a recent study, overexpression of the *M. javanica* FAR effector (mj-far-1) rendered plants more susceptible to nematodes infection^25,26^. One of the universal plant-defense mechanisms upon pathogen infection is the production of a wide variety of compounds collectively termed oxylipins. Oxylipins are a large family of oxidized polyunsaturated fatty acids distributed throughout the plant and animal kingdoms. These highly dynamic metabolites serve as signaling molecules and regulate many different biological functions, such as stress and developmental processes, and contribute to the plant's innate immune response directly as antimicrobial and/or antifungal factors, or indirectly as secondary messengers, to modulate the plant–pathogen interaction^27–29^. The biosynthesis of plant oxylipins is initiated by the release of linolenic or linoleic acids from cell membranes, which are converted to fatty acid hydroperoxides either through an enzymatic pathway by the action of 9-/13-lipoxygenases (9-/13-LOX), α dioxygenase (α-DOX), or a non-enzymatic pathway in the presence of singlet oxygen^30–32^. The hydroperoxide products serve as substrates for at least six alternative enzymatic pathways, resulting in the generation of hundreds of different oxylipin molecules with diverse structures and functions, classified as hydroperoxides, hydroxides, ketotrienes, ketodienes, epoxides and diols, trials, dicarboxylic acids, and ketols, among others. Among the oxylipins, the jasmonate (JA) group has been well-characterized and is shown to mediate plants’ defense response against necrotrophic pathogens and herbivores^33,34^ .While the functions of the vast majority of oxylipins have yet to be elucidated, their production has been shown to change dramatically upon microbial and fungal pathogen invasion, suggesting an important role in defense^35–38^. Deficiencies in the LOX pathway lead to alterations in the plant’s response to pathogen attack, supporting the prominent role of oxylipins in the establishment of resistance^39^. Oxylipins synthesized via the 9-LOX pathway are involved in plant development, including root architecture, senescence and seed germination, and in the stimulation of plant defense upon pathogen attack. The 9-LOX derivatives are among the most active oxylipins in terms of antimicrobial and/or antifungal activity and, in their ability to regulate the programmed cell death responses ^40–45^.

Recent findings have implicated lipid metabolic pathways in the defense responses of plants to RKN^46–48^. For example, microarray-based expression profiling indicated that several fatty acid metabolism genes, including *LOX*, patatin-like protein 1 (*PLP1*), and 12-oxophytodienoate reductase (*OPR*), are induced in susceptible soybean roots in response to soybean cyst nematode infection^49^. Similarly, LOX activity increased in pea root^50^ and soybean roots^49^ upon infection with cyst nematodes. Moreover, degradation of trophic cells, indicating a hypersensitive response, is accompanied by increased activity of pea LOX^51^. Genetic evidence of the involvement of the maize 9-LOX-biosynthesis pathway in resistance mechanisms to RKN was further provided by the analysis of the *lox3*-knockout mutant of maize, which displayed increased attraction and susceptibility to RKN compared to a near isogenic wild-type line^52^. Similarly, Ozalvo et al.^25^ showed that in *Arabidopsis*, the 13-LOX enzyme, LOX4, operates as a root-specific inducer of major defense signaling pathways in response to nematode infection. Recently, a wide screening of oxylipins against *M. javanica* second-stage juveniles (J2) showed that (9S,10E,12Z,15Z)-9-hydroxy-10,12,15-octadecatrienoic acid (9-HOT) application attenuates nematode viability^53^. 9-HOT is produced by the 9-LOX pathway, controlling root development and inducing cell-wall defense responses such as callose accumulation and reactive oxygen species production^40^. We hypothesize that the dynamics of host lipid-mediated signaling induces changes in *M. javanica* infective juveniles' gene-expression profile associated with parasitism. In this study, we report the first comprehensive oxylipin profiling of tomato roots (*Solanum lypopersicum* cv. Avigail 870) challenged by *M. javanica*, using LC–MS/MS, to provide novel insight into the plant response involving fatty acid-derived molecules upon RKN attack. By using RNA-Seq transcriptome analysis of *M. javanica* exposed to the oxylipin 9-HOT and tomato protoplasts, we identified novel potential *M. javanica* transcripts and effectors which might be classified as early mediators regulating nematode parasitism.

## Results and discussion

### Oxylipin biosynthesis is rapidly induced upon tomato root infection by *M. javanica*

**Figure 1:**
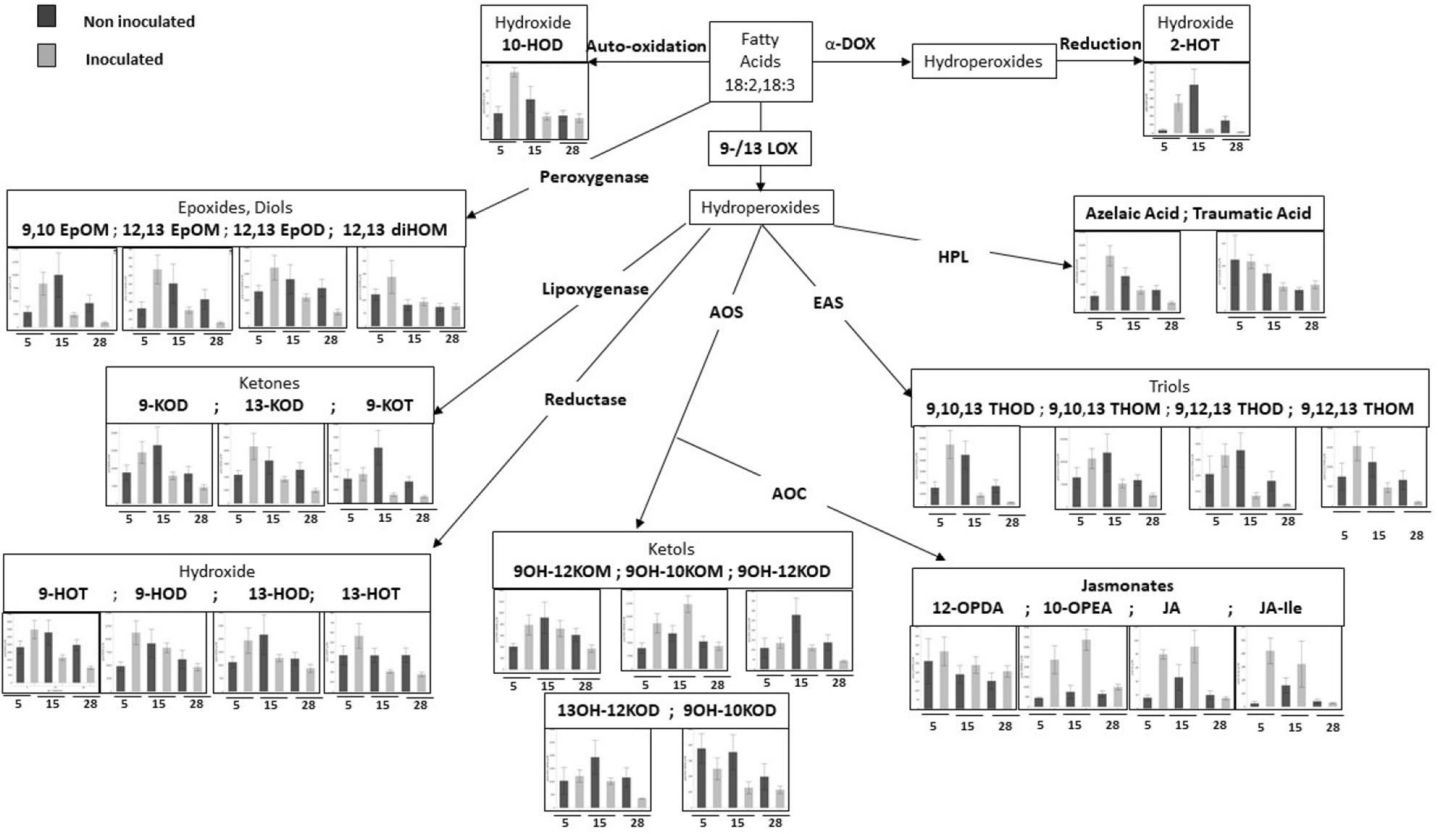
Integrated analysis of the oxylipin profile of tomato roots that were not inoculated (dark gray) or inoculated with *M. javanica* juveniles (light gray) and collected at different time points (5, 15 and 28 DAI). Abbreviations of oxylipins used in this scheme: 18:2, linoleic acid; 18:3, linolenic acid; 2-HOT, 2(R)-hydroxy-9(Z),12(Z),15(Z)-octadecatrienoic acid; 9,10,13-THOD, 9(S),12(S),13(S)-trihydroxy-10(E),15(Z)-octadecadienoic acid; 9,10,13-THOM, 9(S),12(S),13(S)-trihydroxy-10(E),15(Z)-octadecatrienoic acid; 9,12,13-THOD, 9(S),12(S),13(S)-trihydroxy-10(E)-octadecenoic acid; 9,12,13-THOM, 9(S),12(S),13(S)-trihydroxy-10(E),15(Z)-octadecadienoic acid; 12-OPDA, 12-oxo-10,15(Z)-phytodienoic acid; 10-OPEA, 10-oxo-11-phytoenoic acid; JA, jasmonic acid; JA-Ile, jasmonic acid-isoleucine; 9OH-12KOM, 9-hydroxy-12-oxo-octadecaenoic acid; 9OH-10KOM, 9-hydroxy-10-oxo-octadecaenoic acid; 9OH-12KOD, 9-hydroxy-12-oxo-octadecadienoic acid; 13OH-12KOD, 13-hydroxy12-oxo-octadecadienoic acid; 9OH-10KOD, 9-hydroxy-10-oxo-octadecadienoic acid; 9-HOT, 9(S)-hydroxy-10(E),12(Z),15(Z)-octadecatrienoic acid; 9-HOD, 9(S)-hydroxy-10(E),12(Z)-octadecadienoic acid; 13-HOD, 13(S)-hydroxy-9(Z),11(E)-octadecadienoic acid; 13-HOT, 13(S)-hydroxy-9(Z),11(E),15(Z)-octadecatrienoic acid; 9-KOD, 9-keto-10(E),12(Z)-octadecadienoic acid; 13-KOD, 13-keto-9(Z),11(E),15(Z)-octadecatrienoic acid; 9-KOT, 9-keto-10(E),12(Z),15(Z)-octadecatrienoic acid; 9,10 EpOM, 9(R),10(S)-epoxy-12(Z)-octadecenoic acid; 12,13 EpOM, 12(R),13(S)-epoxy-9(Z)-octadecenoic acid; 12,13 EpOD, 11(S),12(S)-epoxy-13(S)-hydroxy-9(Z),15(Z)-octadecadienoate; 12,13 diHOM, (±)-threo-12,13-dihydroxy-9(Z)-octadecenoic acid; 10 HOD, (8E,12Z)-10-hydroxy-8,12 octadecadienoic acid.

To evaluate alterations in oxylipin-mediated defense pathways during tomato root infection by the RKN *M. javanica*, we performed a quantitative analysis of tomato root oxylipins at 5, 15 and 28 days after nematode inoculation (DAI) corresponding to the time of feeding-site formation by infective juveniles, nematode development into J3–J4 stages, and female maturation, respectively as well as of noninoculated roots at the same time points to assess changes in oxylipins accompanying nematode parasitism. A comprehensive oxylipin metabolomic analysis was conducted using the LC–MS/MS platform for quantitative evaluation, and approximately 100 oxylipins were identified (Fig. 1). As shown, inoculated root tissues exhibited an evident alteration in most of the measured oxylipins upon inoculation with RKN J2 compared to noninoculated roots, the latter generally exhibiting no significant changes (Table 1). For oxylipins originated from the LOX pathway—the ketones 13-keto-9(Z),11(E)-octadecadienoic acid (13-KOD) and 9-keto-10(E),12(Z),15(Z)-octadecatrienoic acid (9-KOT), as well as from the reductase pathway—the hydroxides 9-HOT and 13-HOT, we observed a sharp accumulation 5 DAI, followed by a lesser accumulation 28 DAI. Oxylipins of the epoxy alcohol synthase pathway, generating the triol group, followed a similar trend, where 9,10,13-trihydroxy-11(E)-octadecenoic acid (9,10,13-THOD), 9(S),12(S),13(S)-trihydroxy-10(E)-octadecenoate (9,12,13-THOD), 9,10,13-trihydroxyoctadecenoic acid (9,10,13-THOM) and 9,12,13-trihydroxyoctadecenoic acid (9,12,13-THOM) were upregulated at the early time point and gradually decreased as infection proceeded. The presence of oxylipins of the HPL(Hydroperoxide lyase) pathway—azelaic acid and traumatic acid—was measured as well; a significant increase was only observed for azelaic acid 5 DAI compared to noninoculated roots, followed by a clear reduction 15 and 28 DAI. A similar pattern of induction 5 DAI was detected with the α-DOX product 2(R)-hydroxy-9(Z),12(Z),15(Z)-octadecatrienoic acid (2-HOT). Measurement of oxylipins of the 9-allene oxide synthase (9-AOS) pathway—10-oxo-11-phytoenoic acid (10-OPEA) produced from C18:2, and 9-hydroxy-10-oxo-octadecaenoic acid (9OH-10KOM) and 9OH-12KOM belonging to the ketol-group products of auto-oxidation of allene oxides, demonstrated increased accumulation 5 DAI which was sustained 15 DAI (Fig. 1). Among the JA, increased accumulation of JA and JA-Ile was observed 5 DAI compared to noninoculated root tissues, along with a gradual decrease as the infection proceeded. Evidence for the functional role of JA in regulating plant defense response to nematode infection is controversial^52,54–58^. To settle this conflict, a recent study by Gleason et al.^59^ has brought new insight to this issue, positioning the JA precursor 12-OPDA, but not JA/JA-Ile, as a key defense signaling molecule involved in regulating plant susceptibility to nematodes^59^. Our results indicate that while JA and JA-Ile were induced 5 and 15 DAI during compatible interactions with the nematodes, 12-OPDA levels were not affected by nematode infection at the tested time point. In Gleason et al.^59^ study, OPDA accumulation was already detected within 2 DAI. In our study, the earliest time point of 5 DAI might therefore have been too late for detecting differences in 12-OPDA levels. Taken together, our results put several oxylipins, among them the hydroxides, triols, ketones, epoxides, ketols and the JA group at the interface during the early time point of the parasitic interaction.

**Table 1:** Summary of oxylipin concentrations in tomato roots that were or were not inoculated with *M. javanica*, at different time points (5, 15 and 28 DAI).

Oxylipin (pmol/g FW)	Inoculated			Non inoculated		
	5	15	28	5	15	28
10HOD	27.37^A^*^a^	9.67^B^*	8.99^B^*	10.91^b^	16.51	9.99
10OPEA	2364.67^ABa^	3369.57^Aa^	993.12^B^	443.02^b^	746.49^b^	645.96
12,13-diHOM	188.45	93.38	76.93	121.57	81.76	73.54
12,13-EpOD	2236.16^A^	1105.21^B^	542.14^Bb^	1329.46	1796.84	1457.07^a^
12,13-EpOM	669.09^A^**	204.16^B^**	61.94^B^**	223.67	507.81	327.39
12OPDA	316.06	240.42	206.3	262.34	189.08	153.09
13HOD	1937.49^A^	1269.64^AB^	879.52^B^	1113.85	2160.01	1245.95
13HOT	565.65^A^	205.65^B^	175.4^Bb^	369.25	367.6	370.02^a^
13KOD	4309.8^A^	1831.64^B^	976.2^Bb^	2167.59	3241.47	2544.46^a^
13OH-12KOD	1208.85^A^	1013.99^A^	354.13^B^	1028.73	1929.98	1154.71
2HOT	344.74^A^**^a^	38.06^B^**	11.83^B^**^b^	56.13^b^	346.77	143.51^a^
9,10,13-THOD	54,052.1^A^**^a^	8801.4^B^**^b^	2476.4^B^**^b^	15,548.7^b^	39,197.7^a^	16,927.4^a^
9,10,13-THOM	107140^A^**	51751^B^**	26722^B^**^a^	65,231	103,095	59332^b^
9,10-EpOM	8339.47^A^**^a^	2385.1^B^**	872.46^B^**	2900.32^b^	8196.66	4581.32
9,12,13-THOD	3272.46^A^**	726.85^B^**	175.9^B^**^b^	2084.84	2930.54	1652.37^a^
9,12,13-THOM	15363^A^**	4670.7^B^**	963.1^B^**^b^	7470.75	9185.91	6605.78^a^
9HOD	11,215.8^Aa^	8343.5^AB^	4649.6^B^	4812.66^b^	7774.09	6130.96
9HOT	3477.26^A^**	1645.98^B^**	985.45^B^**^b^	2322.58	3290.39	2468.77^a^
9KOD	14,491.3^A^**	7874.9^AB^**	4639.1^B^**	8778.1	13,991.5	8500.3
9-KOT	2198.54^A^**	624.23^B^**^b^	476.77^B^**^b^	1838.94	3537.96^a^	1632.98^a^
9OH-10KOD	248.4	127.73	114.7	377.555	312.543	197.694
9OH-10KOM	8693.3^ABa^*	12,345.2^Aa^*	4330.9^B^	3947.57^b^*	5668.78^b^*	5221.29
9OH-12KOD	134.34^A^	110.15^A^	42.54^Bb^	108.79	229.74	137.02^a^
9OH-12KOM	786.13	722.36	368.27	404	758.67	609.47
Azelaic acid	8375.7^A^**^a^	3074.29^B^**	1180.13^B^**^b^	2214.3^b^	4460.46	3127.14^a^
Jasmonic acid (JA)	79.48^A^**^a^*	90.88^A^**	15.1^B^**	15.67^b^*	37.89	19.81
JA-Ile	419.77^a^	320.28	28.25	24.23^b^	131.93	40.87
Traumatic acid	109.4^A^**	53.39^B^**	57.69^B^**	113.41	71.88	45.85

Data were analyzed by one-way ANOVA (*p* < 0.05), and compared by Student t-test to test for significant differences between inoculated and noninoculated values at the same time point, and Tukey HSD test for statistical significance between different time points for the same treatment. Uppercase letters refer to significant differences between means of the different time points in the same treatment; lowercase letters refer to significant differences between means of the same time point in different treatments. *p < 0.0001, **p < 0.001.

### *M. javanica* infection induces expression of tomato oxylipin-biosynthesis genes *LOX1.2*, *AOS1*, *OPR2* and *α-DOX1*

**Figure 2:**
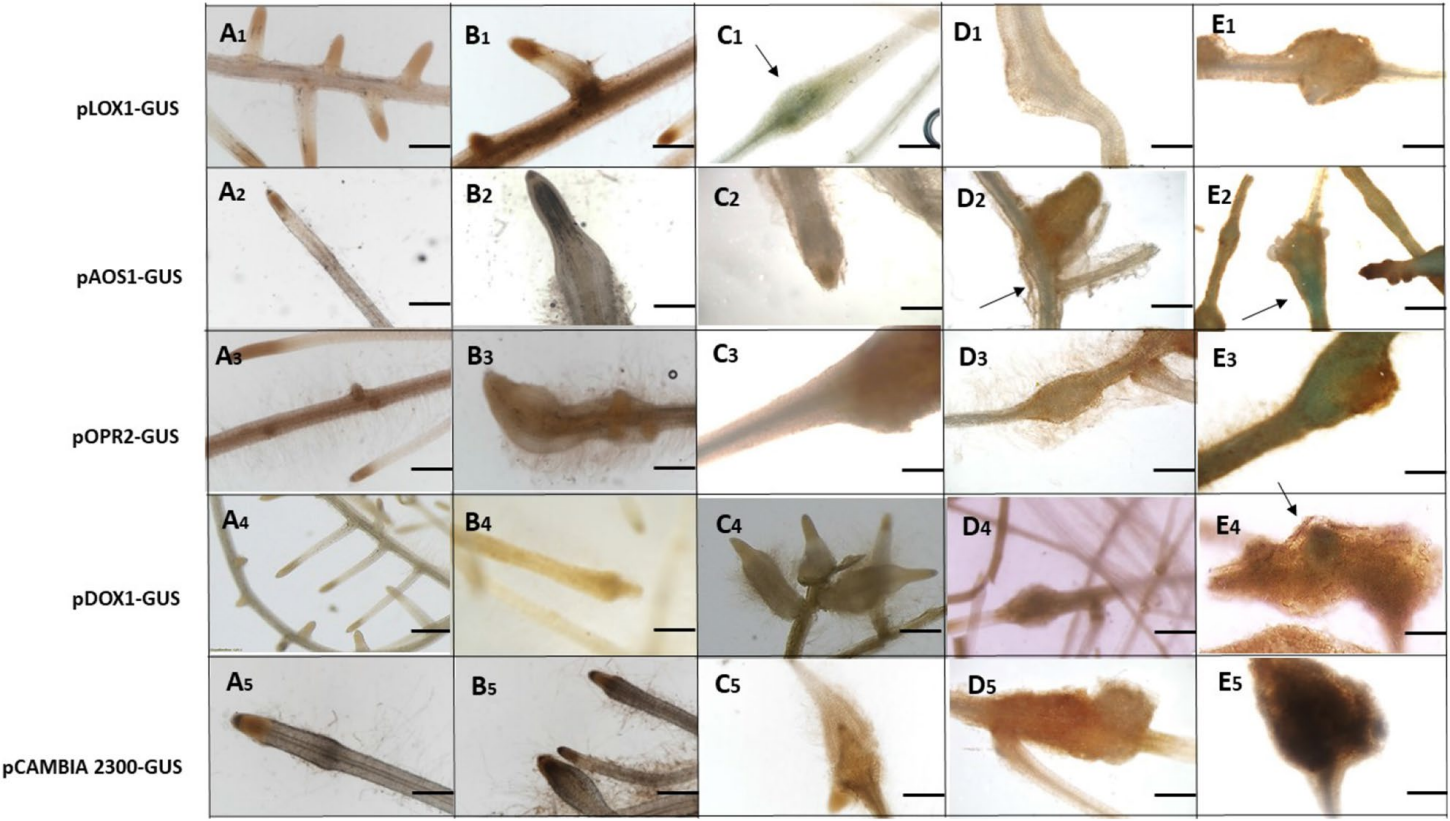
Microscopic analysis of GUS expression patterns in root-knot nematode-inoculated tomato roots harboring *LOX1.2, AOS1, OPR2 and, α-DOX1* promoter: GUS fusion constructs. (**A1–E1**) Micrographs of *LOX1.2*–GUS reporter line. (**A1**) Noninoculated root harboring the *LOX1.2*:GUS fusion construct exhibits no GUS signal related to root tip or elongation zone. (**B1**) Roots 2 days after inoculation (DAI). (**C1**) Inoculated roots 5 DAI. (**D1**) Developing galls 15 DAI. (**E1**) Mature galls 28 DAI. (**A2–E2**) Micrographs of AOS1–GUS reporter line. (**A2**) Noninoculated root harboring the *AOS1*:GUS fusion construct exhibits no GUS signal related to root tip or elongation zone. (**B2**) Roots 2 DAI. (**C2**) Inoculated roots 5 DAI. (**D2**) Developing galls 15 DAI. (**E2**) Mature galls 28 DAI. (**A3–E3**) Micrographs of OPR2–GUS reporter line. (**A3**) Noninoculated root harboring the *OPR2*:GUS fusion construct exhibits no GUS signal related to root tip or elongation zone. (**B3**) Roots 2 DAI. (**C3**) Inoculated roots 5 DAI. (**D3**) Developing galls 15 DAI. (**E3**) Mature galls 28 DAI. A5–E5, Micrographs of αDOX1–GUS reporter line. (**A4**) Noninoculated root harboring the αDOX1:GUS fusion construct exhibits no GUS signal related to root growth. (**B4**) Roots 2 DAI. (**C4**) Inoculated roots 5 DAI. (**D4**) Developing galls 15 DAI. (**E4**) Mature galls 28 DAI. (**A5–E5**) Micrographs of pCAMBIA2300–GUS reporter line; empty pCAMBIA2300 vector fused to GUS reporter served as a control. (**A5**) Noninoculated root harboring the pCAMBIA2300*:*GUS fusion construct exhibits no GUS signal related to root tip or elongation zone. (**B5**) Roots 2 DAI. **C5** Inoculated roots 5 DAI. (**D5**) Developing galls 15 DAI. (**E5**) Mature galls 28 DAI. Arrows indicate GUS staining (C1, D2, E2, E3, D4 and E4): Micrographs viewed under a light microscope. Bright-field image of roots and galls photographed through a stereomicroscope. Bars: **A1–A5, B1, B4, B5, C1, C4–C5, D3–D5** = 50 μm; **B2–B3, C2–C3, D1–D2, E1–E5** = 500 μm.

To correlate oxylipin occurrence with genetic biosynthetic pathways, we used the GUS–promoter bioassay for spatial and temporal expression of oxylipin-biosynthesis genes *LOX1.2*, *AOS1, OPR2* and *αDOX1*. Transgenic tomato hairy root lines (line 870) carrying pLOX1.2–GUS, pAOS1–GUS, pOPR2–GUS and pα-DOX1-GUS constructs, and a negative control consisting of hairy root lines carrying empty pCAMBIA2300 vector, were tested for GUS activity at 2, 5, 15 and 28 DAI compared to noninoculated transgenic tomato hairy root lines and a negative control (Fig. 2). LOX's involvement in the plant response to RKN has been previously reported^46–48^. *LOX1.2* is a 9-LOX gene (solyc01g099210.2.1) that is homologous to *LOX1* in *Arabidopsis*, located in the cytosol and known to catalyze the hydroperoxidation of linoleic acid, and thus be involved in the pathway of 9-oxylipin biosynthesis (UniProtKB—P38416, LOXB_SOLLC). *LOX1.2* expression was observed as a mild signal in the vascular system of the forming gall 5 DAI (Fig. 2C1), with only a faint signal observed 15 DAI and 28 DAI (Fig. 2D1,E1). These results are in good agreement with the oxylipin analysis where we observed that the 9-LOX products, including azelaic acid, a mobile product of HPL that primes systemic acquired resistance^60^, were elevated 5 DAI and downregulated 28 DAI. The ketones synthesized by LOX, 9-KOT and 13-KOD, as well as through hydroxide synthesis by reductase, 9-HOT, were induced 5 DAI with a clear reduction 28 DAI. Interestingly, these oxylipins have been shown to exert significant nematicidal properties against J2^53^, as well as antimicrobial activity^42^. Among the products of the epoxy alcohol synthase pathway, the trihydroxy oxylipins 9,10,13-THOD, which have been reported to reduce infection by the fungus *Blumeria graminis* in barley, were upregulated 5 DAI, and decreased 15 and 28 DAI. Similarly, 9,12,13-THOD and 9,12,13-THOM, which are known to have antimicrobial activity, were upregulated 5 DAI and downregulated 15 and 28 DAI^38,53,61^.

Next, the expression of *13-AOS1* (solyc04g079730.1.1), located in the thylakoid membrane and involved in JA biosynthesis (UniProtKB—K4BV52, AOS1_SOLLC), was studied. *AOS1* reporter lines exhibited GUS staining in the vascular cylinder attached to the developing gall 15 DAI (Fig. 2D2), which strongly increased by 28 DAI in the developed gall (Fig. 2E2).

Functional analysis of AOS1 in rice indicates that rice plants overexpressing *AOS1* are less susceptible to *M. graminicola*^62^. Similarly, *Arabidopsis thaliana AOS* mutant *dde2* shows more galling by *M. hapla* than the wild type^59^. However, being a wound-inducible gene^63,64^, *AOS1* expression at this late time point might indicate that a localized wound response is induced by the developing nematode^65^. *OPR2* (solyc01g0103390.2.1) is known to be weakly expressed in roots and to be involved in the oxylipin-biosynthesis pathway, but as it belongs to the type 1 subfamily, this isoform is not predicted to be a major contributor to JA biosynthesis^66,67^ (UniProtKB—Q9FEX0, OPRL_SOLLC). The RKN-inoculated *OPR2* promoter–GUS reporter line only showed a strong GUS signal 28 DAI in mature galls Fig. 2E3). Strassner et al.^66^ demonstrated that in contrast to *Le*OPR3, wounding does not induce *Le*OPR1 or *Le*OPR2 expression and that *Le*OPR2 is expressed at comparatively low levels in tomato roots, leaves, and flowers. However, GUS detection within the mature gall implicated *Le*OPR2 in a late response, although the physiological function in the mature gall remains obscure. α-DOX1 (solyc02g087070) is a fatty acid-hydroperoxidase located in the endomembrane system, involved in many processes, such as response to oxidative stress, lipid metabolic processes and a salicylic acid (SA) stimulus (UniProtKB—Q69F00 (Q69F00_SOLLC))^36,68–70^. Similarly, α-DOX1 exhibited GUS staining associated with the adult nematode within the developed gall 28 DAI (Fig. 2E4). No staining was observed in the negative controls (Fig. 2A5–E5). α-DOX1 has been previously detected in tomato and *Arabidopsis* roots tissues and its generated oxylipins have been suggested to mediate the response of roots to several environmental stresses^68,70^. Our analysis indicated that α-DOX1 is expressed within the mature gall. 2-HOT, the main product of α-DOX, which has been shown to be upregulated by bacterial inoculation and herbivore infestation^61,71^, was found to be significantly upregulated by RKN inoculation 5 DAI. The lack of synchronized expression of the biosynthesis genes and their accumulated products at early time points, observed for OPR2 and α-DOX1, might be explained by a low level of expression requirement or much earlier expression occurring upon infection. Overall, multiple expression patterns of oxylipins derived from the diverse branched pathways might be the result of plant immunity and/or induced by the nematode for successful establishment. As noted by Prior et al.^72^ and Iberkleid et al.^73^, nematodes' FAR protein binds to linolenic and linoleic acids, which are precursors of the oxylipin molecules, and have further been found to inhibit LOX-mediated products. Our results indicate that upregulation of oxylipin biosynthesis is a primary response to nematode inoculation, and as such must be counteracted by an adequate nematode defense response.

### RNA-seq analysis of *M. javanica* J2 following exposure to 9-HOT and tomato protoplasts

To identify transcripts and effectors that are subject to plant host lipid-signaling regulation, the oxylipin 9-HOT, which—among other oxylipins—is induced in tomato roots by RKN infection (Fig. 1), was chosen as the inducer. Similarly, in an attempt to mimic exposure to endogenous metabolites secreted within plant tissue during the parasitic stage, J2 were exposed to tomato protoplasts. Freshly hatched J2 incubated in MES buffer or MES + ethanol served as controls for tomato protoplasts and the studied oxylipin, respectively. Altogether, eight cDNA libraries were constructed, generating a total of 170,786,458 reads, 121,161,460 reads after quality and adaptor trimming by Trimmomatic version 0.35^74^. The eight libraries represented: (1) biological duplicates of freshly hatched J2 with 15,208,788 reads; (2) biological duplicates of J2 exposed to protoplasts for 3 h with 15,572,666 reads; (3) biological duplicates of J2 exposed to 9-HOT for 3 h with 14,677,503 reads; (4) biological duplicates of J2 in MES + ethanol with 15,121,772 reads. All RNA-Seq raw data reads were uploaded to NCBI under BioProject Accession PRJNA480605. From the 112,732,357 high-quality paired-end reads, 72.01% of the freshly hatched J2, 67.11% of the J2 exposed to protoplasts, 52.78% of the J2 exposed to 9-HOT and 73.01% of the J2 exposed to MES + ethanol were mapped to the reference genome of *M. javanica*, available on WormBase ParaSite BioProject PRJEB8714 and sequenced by Blanc-Mathieu et al.^75^ (Table 2).

**Table 2:** Summary of statistics for individual RNA-Seq paired-end reads and library.

Biological assay	Library name	No of paired-end reads	No of paired-end reads after quality and trimming	% paired-end reads mapped to *Meloidogyne_javanica* transcriptome (%)
(1)	Protoplasts	21,228,091	13,070,602	73.42
(2)	Protoplasts	21,776,598	18,074,731	60.81
(1)	9-HOT	20,313,528	14,682,317	53.40
(2)	9-HOT	21,446,366	14,672,689	52.17
(1)	MES buffer + Ethanol	23,253,618	16,242,352	74.07
(2)	MES buffer + Ethanol	21,049,270	14,001,192	71.96
(1)	J2	19,814,441	14,227,256	72.44
(2)	J2	21,904,546	16,190,321	71.59

### Uncovering transcriptomic changes in *M. javanica* J2 upon exposure to oxylipin 9-HOT and other plant signals

**Figure 3:**
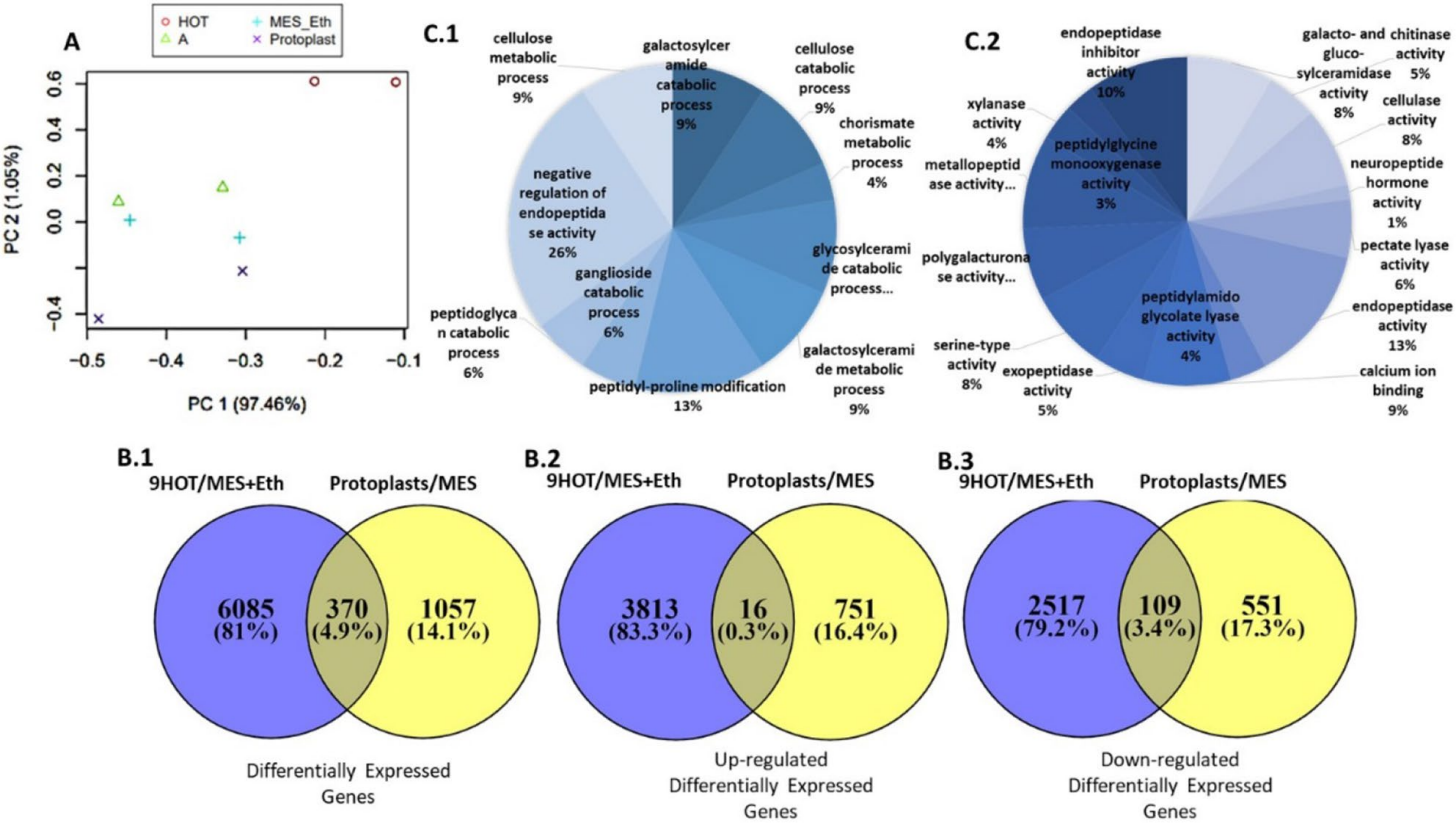
(**A**) Principal components analysis (PCA). Distribution of differentially expressed genes. Three-dimensional representation according to PCA of the differentially expressed genes among the four treatments used in the RNA-Seq analysis: J2 of *M. javanica* exposed to protoplasts, 9-HOT, or MES + ethanol (MES + Eth), or freshly hatched J2. Samples with similar expression profiles lie closer to each other than those with dissimilar profiles. Axes 1 and 2 show robust class separation into two major groups: (1) 9-HOT, (2) J2 and MES + ethanol and protoplasts. (**B**) Venn diagram showing number of overlapping and non-overlapping differentially expressed *M. javanica* genes following exposure to: protoplasts/control (Cont.) and 9-HOT/control. (**B.1**) distribution of all differentially expressed genes. (**B.2**) Distribution of up-regulated genes found in each treatment as well as genes overlapping between both treatments. (**B.3**) Distribution of down-regulated genes found in each treatment as well as genes overlapping between both treatments. Fold change with an absolute value > 2 and < − 2 and *p* value ≤ 0.001 was used for the analyses. (**C**) Gene ontology (GO) annotations of differentially expressed genes of *M. javanica* J2 exposed to 9-HOT vs. control unigenes at multilevel using BLAST2go software. The GO terms were categorized into (**C.1**) biological process and (**C.2**) molecular function. Pie chart slices represent the percentages of genes identified in a particular category among the differentially expressed genes.

To measure transcript regulation of *M. javanica* J2 by 9-HOT and plant signals, we measured changes in gene expression of infective J2 exposed to tomato protoplasts compared to their control (MES), and of infective J2 exposed to 9-HOT compared to their control (MES + ethanol). Statistical analysis of the differentially expressed genes (DEG) was performed using the DESeq2 package^76^. The threshold for DEG was FDR ≤ 0.001 and log2 fold change (FC) smaller than − 2 or greater than 2. Overall 7530 DEG were identified among the treatments—J2 exposed to protoplasts and J2 exposed to 9-HOT—compared to their respective controls. Principal component analysis (PCA) was conducted to determine and visualize the significant correlation between the different treatments using R (version 3.0.0) (http://www.R-project.org) and the FactoMineR R package^77^. PCA of the transcriptomic data was performed for all expressed gene profiles (Fig. 3A). The two dimensions made up 98.51% of the total variance, indicating that most of the factors included in the data were responsible for the significant variation in DEG between treatments. Dimension 1 accounted for 97.46%, and dimension 2 for 1.05% of the variation. The first and second PC axis separated the freshly hatched J2 exposed to 9-HOT from all other groups. Taken together, these results suggest that the different treatments can be divided into two major expression profiles of DEG: (1) freshly hatched J2, J2 exposed to protoplasts and MES + ethanol and (2) J2 exposed to 9-HOT, the latter demonstrating the most variation (Fig. 3A).

Next, all 7512 DEG were subjected to Venn diagram analysis illustrating DEG distribution among treatments (Fig. 3B). A total of 6085 DEG (81%) and 1057 DEG (14.1%) were found to be expressed exclusively in the 9-HOT and protoplast treatments, respectively, and 370 DEG (4.9%) were expressed in both treatments (common DEG) (Fig. 3B.1). A total of 4580 DEG were found to be upregulated (83.3%), with 3813 (83.3%) and 751 (16.4%) expressed in the 9-HOT and protoplast treatment, respectively, and 16 (0.3%) expressed in both treatments (Fig. 3B.2). GO enrichment analysis of the annotated DEG revealed several enriched biological processes (Fig. 3C.1) and molecular functions (Fig. 3C.2). Detailed analysis of identified DEG following exposure to protoplast and 9-HOT revealed that the key enriched biological processes were negative regulation of endopeptidase (GO:0061135), peptidyl-proline modification (GO:0031543), glycosylceramide catabolic process (GO:0004348), cellulose metabolic process (GO:0030243), galactosylceramide metabolic process (GO:0006683). The key enriched molecular functions included polygalacturonase activity (GO:0004650), calcium ion binding (GO:0005509), pectate lyase activity (GO:0030570), endopeptidase activity (GO:0004175), cellulose activity (GO:0030243; 0030245), and endopeptidase inhibitor activity (GO:0004866) (Fig. 3C).

To further classify the observed changes, all 7512 DEG were analyzed for Pathway enrichment using the webserver of KOBAS 3.0^78^ KEGG, GO and PANTHER database enrichment analysis. When compared to *Loa loa* for glutathione metabolism (loa00480), which plays important roles in antioxidant defense^79^, 15 DEG were upregulated and 7 DEG downregulated, for a total of 22 out of 25 known genes involved in this pathway (Fig. S1A). In the Wnt signaling pathway (loa04310), known to regulate crucial aspects of cell-fate determination during embryonic development^80^, 24 DEG were upregulated and 5 DEG were downregulated—29 out of the 65 known genes represented in this pathway (Fig. S1B). In the fatty acid biosynthesis pathway (loa00061), which is a precursor for a variety of important building blocks^21,81,82^, 8 DEG were upregulated , 10 out of the 10 known genes. For retinol metabolism (loa00830), a total of 6 DEG were upregulated out of 6 known genes in this pathway. All of these DEG were enriched following exposure to 9-HOT. Following exposure of J2 to protoplast treatment, the calcium signaling pathway (loa04020) was represented by 27 upregulated DEG out of a total of 57 genes known to be involved in this pathway. In the inositol phosphate metabolism pathway (loa00562), 9 DEG were upregulated out of a total of 38 known genes in this pathway. In the phosphatidylinositol signaling system (loa04070), 9 DEG were upregulated out of 57 known genes in this pathway.

### 9-HOT application regulates the expression of genes encoding carbohydrate-active enzymes (CAZymes) related to cell wall modification and degradation

To further evaluate the effect of 9-HOT and protoplasts on CAZymes, we investigated families of structurally related catalytic and carbohydrate-binding modules (CBM) of enzymes that degrade, modify or create glyosidic bonds. We focused on the DEG encoding CAZymes related to cell wall biosynthesis, modification and remodeling^82^ (Fig. S2). Differential expression of four categories of CAZYmes were represented following exposure to 9-HOT and protoplasts, i.e., genes encoding carbohydrate esterase (CE), glycoside hydrolase (GH), glycosyl transferase (GT) and polysaccharide lyase (PL) (Figure S2A). Among the CAZyme categories involved in cellulose degradation (Fig. S2B), two families of GH were differentially expressed following protoplast and 9-HOT treatments. These included GH5 and GH7 presented by endo-1,4-β-glucanase/cellulase and β-glucosylceramidase and chitosanase. Among the hemicellulose-degrading genes (Fig. S2C), two categories were represented by GH31 and CE1, such as α-galactosidase, α-mannosidase, glucosyltransferase acetyl transferase and carboxylesterase.

### 9-HOT induces major differences in nematode's effector-encoding gene expression

**Figure 4:**
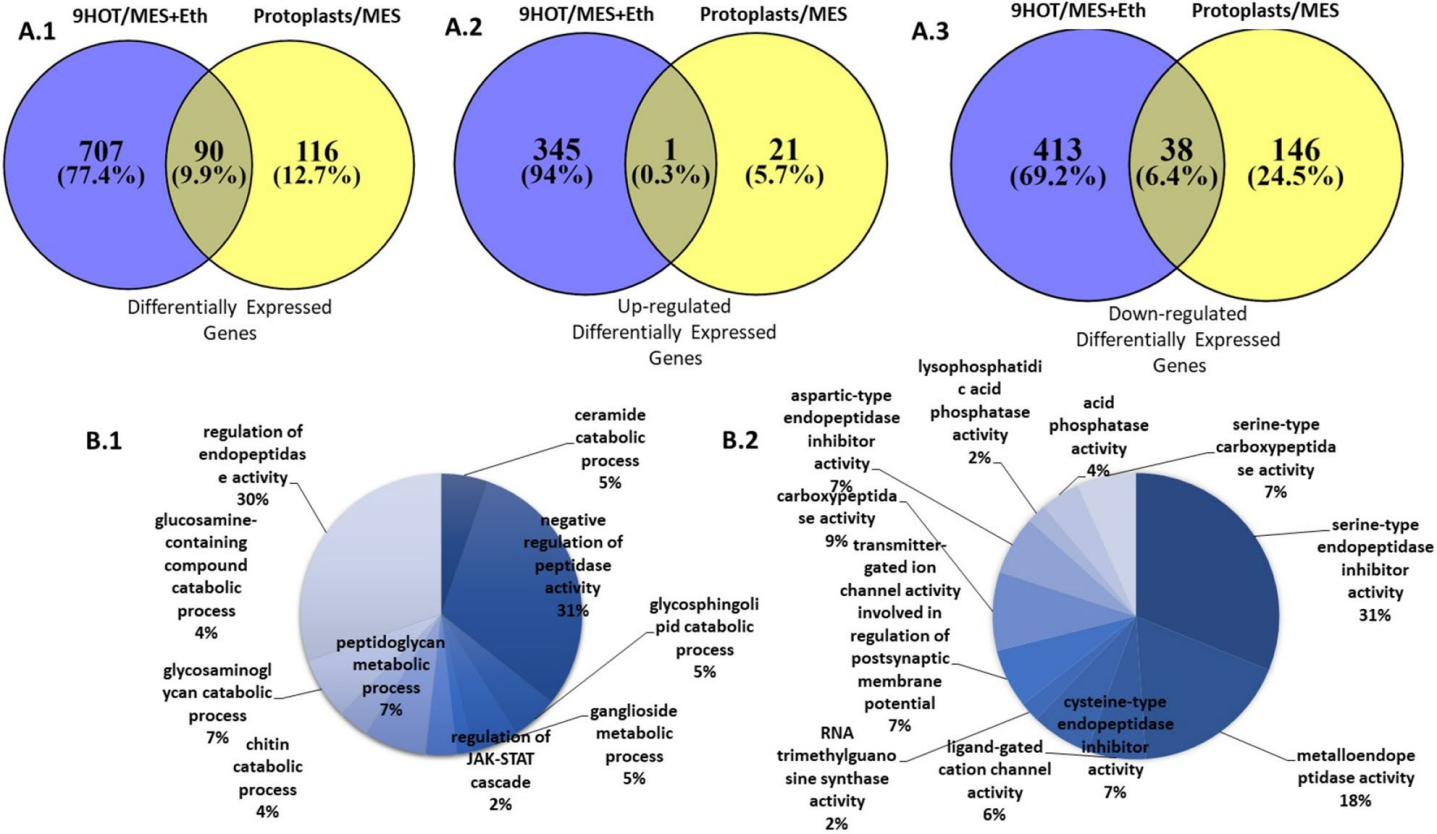
(**A**) Venn diagram depicting the distribution of DEG including Signal Peptide according to SignalP5.0. (**A.1**) Venn diagram showing the number of overlapping and nonoverlapping total differentially expressed genes of *M. javanica* transcripts following exposure to: protoplasts/control (Cont.) and 9-HOT/control. (**A.2**) Distribution of up-regulated genes found in each treatment as well as genes overlapping between both treatments. (**A.3**) Distribution of down-regulated genes found in each treatment as well as genes overlapping between both treatments. Fold change with an absolute value > 2 and < − 2 and *p* value ≤ 0.001 was used for the analyses. (**B**) Gene ontology (GO) annotations of differentially expressed genes including Signal Peptide according to SignalP5.0, of *M. javanica* J2 exposed to 9-HOT vs. control unigenes at multilevel using BLAST2go software. The GO terms were categorized into (**B.1**) biological process and (**B.2**) molecular function. Pie chart slices represent the percentages of unigenes identified in a particular category among the differentially expressed genes encoding effectors.

Given that we were interested in genes involved in governing parasitism, i.e., effectors, our next step was an *in-silico* analysis to identify differentially expressed transcripts that might encode secreted effectors. DEG that contained a predicted signal peptide according to SignalP5.0^83^ and which do not carry TMHMM (transmembrane alpha helix motifs) were subjected to Venn diagram analysis illustrating DEG distribution among treatments (Fig. 4A). A total of 913 DEG with a signal peptide were identified (12.2% of total DEG). Among these, 707 DEG (77.4%) and 116 DEG (9.9%) were expressed in the 9-HOT and protoplast treatments, respectively, and 90 DEG (12.7%) were expressed in both treatments (Fig. 4A.1). A total of 367 DEG with a signal peptide were found to be upregulated (33.7%) (Fig. 4A.2), and 597 (54.9%) DEGs were downregulated (Fig. 4A.3).

All DEG that were upregulated by 9-HOT treatment were analyzed for GO terms (Fig. 4B) in each of the three main categories (biological process, molecular function and cellular component classification) of the GO classification. The GO cellular component classification of the DEG indicated 12% of extracellular region and 88% of the membrane part. Biological process was represented by 10 categories: 31% by negative regulation of peptidase activity (GO:0010466), 30% by regulation of endopeptidase activity (GO:0052548), about 4–7% each by chitin, ceramide, glucosamine, glycosaminoglycan and glycolsphingolipid catabolic process (GO:0006032, GO:0046514, GO:1901072, GO:0006027 and GO:0046479, respectively) (Fig. 4B). Molecular function was represented by 11 categories: 31% by serine-type endopeptidase inhibitor activity (GO:0004867), 18% by metalloendopeptidase activity (GO:0004222), 9% by carboxypeptidase activity (GO:0004180). Next, we validated the expression profile of predicted effectors in protoplast- and 9-HOT-treated *M. javanica* J2. For that purpose, seven selected DEG from the *M. javanica* J2 transcriptome were further confirmed and validated by quantitative reverse transcription (qRT)-PCR. Four downregulated genes and three upregulated genes were selected for quantitative analyses, on the basis of being potentially secreted and involved in the pathogenic process and carrying a signal peptide. One of the DEG was found in J2 exposed to protoplasts: protoplasts#1 encoding DB domain-containing protein (M.javanica_Scaff1102g012786); six were found in J2 exposed to 9-HOT: 9-HOT#1—unknown gene (M.javanica_Scaff10526g059067), 9-HOT#2—SCP domain-containing protein (M.javanica_Scaff139g002482), 9-HOT#3—unknown gene (M.javanica_Scaff8981g053951), 9-HOT#4—putative esophageal gland cell secretory protein 3 (M.javanica_Scaff2606g024064), 9-HOT#5—calycin-like domain (M.javanica_Scaff24242g089056), 9-HOT#6—triacylglycerol lipase (M.javanica_Scaff6853g045742), all of which are shown in Fig. S3. For all qRT-PCR analyses, two housekeeping genes were chosen as reference genes for *M. javanica*: endogenous reference genes *18S* and *EF-1α* (Table 1S). For all tested transcripts, our analysis remained similar to the transcriptomic trend of the FC data.

### Differentially regulated effector-encoding genes are implicated in cell wall modifications, stress response, plant immune suppression and nematode development, enabling parasitism

Among the 346 upregulated DEG in the 9-HOT treatment were genes implicated in nematode growth and development, such as *MLT-10*, the cuticlin-1, epicuticulin gene family and collagen, all of which participate in various cellular and developmental processes required for nematode molting and fecundity. Within the root tissues, RKN undergo three molting stages; in each molt, the makeup of the cuticle surface coat's compounds changes, one among many strategies acquired by plant-parasitic nematodes to avoid plant immunity^84^. Similarly, previous studies have shown that hormones and different compounds secreted by the roots trigger changes in the surface cuticle of sedentary plant-parasitic nematodes^10,85^.

In addition, a group of genes implicated in oxidation–reduction activity required for coping with oxidative stress response were induced by 9-HOT (i.e., glutaredoxin, thioredoxin-like domain). Similarly, effectors containing a C-type lectin, which was found to delay the oxidative burst in tobacco leaves following infection by *M. graminicola*^86^, were upregulated following exposure to 9-HOT (*M.javanica*_Scaff6180g042809) and protoplasts (*M.javanica*_Scaff16387g074472) (Fig. 4A). In addition, genes involved in lipid modification (several genes of triacylglycerol lipase, calycin domain) were also differentially expressed following 9-HOT treatment. Differential regulation of several proteases was observed: a carboxypeptidase, serine carboxypeptidase (SCP), was studied in depth and was shown to play a critical role in the development, invasion, and pathogenesis of certain parasitic nematodes and other animal pathogens^87^. Another protease, a serine proteinase, that was induced has also been shown to be involved in mediation of host invasion by the parasitic nematode *Steinernema carpocapsae*^87^. Similarly, alteration in the expression of chorismate mutase (CM) and venom allergen-like protein (Vap2), well-studied effectors that are involved in suppression of defense reactions of the host cell during the infection stages, were detected^14,87–91^. Papain inhibitor, which might be related to pathogen effectors that inhibit apoplastic papain-like cysteine protease (PLCP) which are strongly associated with the effector triggered immunity (ETI) response^92^, were strongly upregulated upon 9-HOT treatment. An extensive representation of genes involved in cell wall modification and remodeling, carrying a signal peptide, were altered upon 9-HOT and protoplast exposure, e.g., effectors carrying a Rare lipoprotein A domain found in several effectors, such as Mc-EXP1, *Gr*EXPB1 and *Gr*EXPB2, associated with cell wall extension in *M. chitwoodi* and *G. rostochiensis*, respectively^93,94^, were upregulated upon 9-HOT application. Additional observed differentially regulated genes were involved in cell wall degradation and modification (Fig. S2), including cellulose binding protein (CBP)—a nematode excretion protein that appears to be associated with the breakdown of cellulose present in the plant cell wall^95,96^—and pectate lyases, known to play a key role in pectin degradation by catalyzing the random cleavage of internal polymer linkages (endopectinases). Similarly, pectate lyases have been isolated from several sedentary plant-parasitic nematodes, such as species of *Heterodera*, *Globodera*, and *Meloidogyne*^97–99^, and have been shown to be released into the plant tissue through the stylet of the nematode. GH family 38 (GH38) α-mannosidase was upregulated by 9-HOT; this protein is involved in α-mannose cleavage, carrying hemicellulose activity, and has been identified in several phytopathogenic nematode species^100^. In addition, several endoglucanases belonging to the GH5 family were differentially expressed upon 9-HOT treatment (Fig. S2); these have been shown to facilitate penetration and migration into root tissue and were localized to the esophageal glands of infective juveniles^95,101,102^. All of these genes are part of a cocktail of cell wall-degrading and modifying enzymes that are thought to soften and degrade the structure of plant cell walls during nematode migration and to facilitate infection^95,103^. Their fluctuation upon 9-HOT and protoplast treatment might indicate tight regulation governed by oxylipin signals, among others.

### Triacylglycerol lipase and MLT10-like, predicted effectors, are exclusively localized to the *M. javanica* esophageal glands

**Figure 5:**
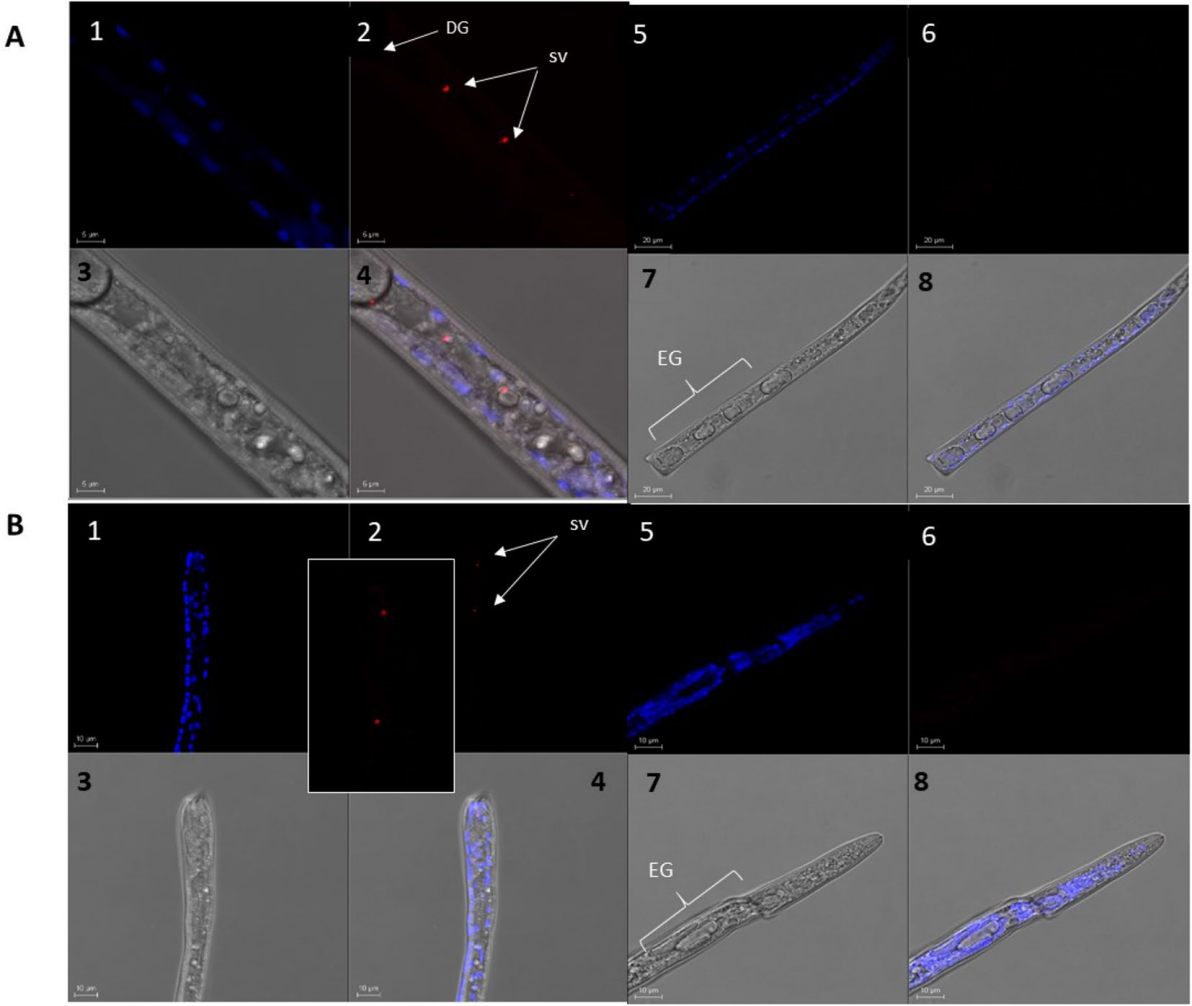
FISH of dissected freshly hatched *M. javanica* J2*. M. javanica* J2 were stained with DAPI (blue) together with (**A**) Triacylglycerol lipase Cy5-specific probe (red). (**A1–4**)** M. javanica** J2 exposed to 9-HOT. Esophageal gland area: D, dorsal gland; SV, subventral glands (combined Z sections). (**A5–8**)** M. javanica** exposed to 0.01 M MES buffer + ethanol; EG, esophageal gland region. **1** and **5** DAPI-stained nuclei (blue) of the dissected nematode as seen from combined sections under fluorescence. **2** and **6** FISH signal of triacylglycerol lipase Cy5-specific probe (red) as seen from combined sections under fluorescence. **3** and** 7** Combined DAPI-stained nuclei (blue) and FISH signal (red) under bright field. **4** and **8 M. javanica** as seen from combined sections under bright field and fluorescence. (**B**) MLT-10 like Cy5-specific probe (red). (**B1–4**)** M. javanica** J2 exposed to 9-HOT. SV, subventral glands (combined Z sections). (**B5–8**)** M. javanica** exposed to 0.01 M MES buffer + ethanol; EG, esophageal gland region. **1** and **5** DAPI-stained nuclei (blue) of the dissected nematode as seen from combined sections under fluorescence. **2** and **6** FISH signal of MLT-10 Cy5-specific probe (red) as seen from combined sections under fluorescence. **3** and** 7** Combined DAPI-stained nuclei (blue) and FISH signal (red) under bright field. **4** and **8 M. javanica** as seen from combined sections under bright field and fluorescence.

Additional effectors that may facilitate plant–nematode interactions through manipulation of the plant defense system, or are required for nematode developmental processes, and which were localized to the esophageal glands upon 9-HOT application, are triacylglycerol (TAG) lipase, which functions in TAG release from lipid droplets by lipolysis in the peroxisome^82^. Interestingly, modulation of plant peroxisomes in giant cells by sedentary RKN has been described previously^104^; and molting cycle MLT-10-like, required for nematode development. Using fluorescence in situ hybridization (FISH), we designed a Cy5-probe to specifically target the spatiotemporal expression of several putative effector-encoding genes derived from the above DEG carrying a signal peptide. FISH results, shown in Fig. 5, localized TAG lipase exclusively to the dorsal and two subventral glands upon J2 exposure to 9-HOT (Fig. 5A1–4), compared to its control (i.e., J2 exposed to MES + ethanol) that showed no fluorescent signal (Fig. 5A5–8). Similarly, MLT-10 was localized to the subventral glands upon J2 exposure to 9-HOT (Fig. 5B1–4), compared to its control (J2 exposed to MES + ethanol) with no fluorescent signal.

These results strengthened our assumption that the DEG containing a signal peptide are potential secreted effectors. Additional analysis of TAG lipase and MLT-10-like by qRT-PCR at different stages of *M. javanica* development further correlated their expression with parasitism (Fig. S4).

## Conclusions

Despite enormous progress in the discovery and identification of nematode effectors in the last decade^7,15,105–110^, less is known about their function and the specific signals required for their induction. Our transcriptomic studies of *M. javanica* provide evidence of transcripts with homology to previously reported plant-parasitic nematode effectors, as well as unknown secreted proteins, all induced by 9-HOT. Together with the oxylipin profile analysis, it seems that oxylipins, while part of the plant's defense response, might play an important signaling role in regulation of the nematode transcriptome. Among the differentially regulated predicted effectors, several were further confirmed by FISH analysis as effectors located within the esophageal glands. Taken together, these results placed oxylipins as early modulators of plant defense signals, as well as important signals regulating nematode parasitism. The implication of G-protein coupled receptor (GPCR) as an oxylipin receptor, shown previously by Affeldt et al.^111^ and Lahvic et al.^112^, should place this group of nematode receptors as important mediators of parasitic behavior. This interaction remains to be studied.

## Methods

### Nematode culture and inoculum preparation

*M. javanica* were multiplied on tomato plants (*Solanum lypopersicum* cv. Avigail 870) in a greenhouse. Nematode egg masses were extracted by cutting roots into pieces and macerating in 0.05% (v/v) sodium hypochlorite (NaOCl) in a blender^113^. The resulting suspension was passed through a set of three sieves (120, 60 and 30 µm). The debris was discarded, while the eggs deposited on the 30-µm sieve were transferred to a 50-mL test tube. Centrifugal flotation with 40% (w/v) sucrose at 6000 rpm for 10 min was performed; the supernatant, containing the eggs, was poured onto a 30-µm sieve and washed with tap water, and eggs were collected in MES buffer^113^ and sterilized as described by van Vuuren and Woodward^114^.

Subsequently, eggs were transferred onto a 30-µm sieve and suspended in 5 mL MES buffer in a petri dish. The petri dish was placed in a growth chamber at 26 °C under dark conditions till hatching (5–6 days)^115^.

### J2 exposure to protoplast treatment

Sterilized tomato seedlings were grown at 26 °C under 16 h daylight in sterile plates on standard-strength Gamborg's B5 Salt medium (DUCHEFA, Haarlem, The Netherlands), supplemented with 2% (w/v) sucrose and solidified with 0.8% (w/v) Gelrite agar (DUCHEFA). Roots were subcultured on the same medium with one root section per petri dish (MINIPLAST, Ein Shemer, Israel) and incubated in a growth chamber at 26 °C in the dark for 2 weeks. Protoplasts were released and isolated from about 40 plates of roots using Demidchik and Tester's^116^ protocol. Fresh protoplasts were incubated with 60,000 freshly hatched sterilized J2 at 26 °C in the dark for 3 h.

### J2 exposure to 9-HOT treatment

The 9-HOT oxylipin used in this study was purchased from CAYMAN CHEMICAL Company, diluted with MES buffer to a final concentration of 10 µM. Freshly hatched sterilized J2 were incubated in vials (500 J2 per vial for a total of 15,000 juveniles for each oxylipin treatment) containing 9-HOT or 0.01 M MES + ethanol (to a final concentration of 10 mM, as a control) for 3 h at 26 °C in the dark.

### cDNA library preparation and high-throughput sequencing

Total RNA extraction from freshly hatched J2, and J2 exposed to protoplasts, 9-HOT and MES + ethanol, was performed with Trizol, using total RNA extraction from the *Caenorhabditis elegans* bioprotocol (2011) with steps 10–12 being replaced by the use of the Arcturus PicoPure RNA Isolation Kit, APPLIED BIOSYSTEMS (Foster City, CA, USA), and stored frozen at − 80 °C till further processing^117^. Quality measurements for total RNA were performed using TapeStation 2200 (Agilent). The RIN (RNA integrity number) values of all 8 samples were between 7 and 10.

### Library preparation and data generation

Eight RNA-Seq libraries were produced using the NEBNEXT ULTRA Directional RNA Library Prep Kit for Illumina (NEB, cat no. E7420) according to the manufacturer's protocol and starting with 100 ng of total RNA. The mRNA pull-up was performed using the Magnetic Isolation Module (NEB, cat no. E7490). The eight libraries were mixed in a single tube at equal molar concentrations. RNA-Seq data were generated using the Illumina NextSeq Machine and Nextera Preparation Kit at the Technion—Institute of Technology in Haifa, Israel. Eight paired-end RNA-Seq libraries of two replicates for each condition were sequenced.

### Differential expression analysis

The sequences were trimmed for adaptor and low-quality sequence removal using Trimmomatic software^74^. Cleaned sequences were mapped using Bowtie2^118^ and quantified using the RSEM method^119^ to the reference genome of *M. javanica* (accession no. GCA_900003945.1). The annotated proteins of the nematode were searched for signal peptides using the software SignalP 5.0^83^.

DEG were identified using the DESeq2 R package^76^. To create the Venn diagrams, we used Venny website http://bioinfogp.cnb.csic.es/tools/venny/^120^. All RNA-Seq datasets were uploaded to the SRA NCBI database under BioProject Accession No. PRJNA480605.

### CAZyme annotation

The search for and functional annotation of CAZymes (automated carbohydrate-active enzyme annotation) was performed using the CAZY database (http://www.cazy.org/) according to Lombard et al.^121^. We assigned the *M. javanica* (accession no. GCA_90003945.1) proteins to the CAZY database using the dbCAN2 meta server (http://bcb.unl.edu/dbCAN2index.php).

### Plant material and growth conditions

Tomato (*Solanum lycopersicon* cv. Avigail 870) seeds were sterilized with 1.4% (v/v) NaOCl for 10 min, washed three times with sterile water for 5 min each, and then planted on standard strength Gamborg's B5 salts medium (DUCHEFA), supplemented with 2% sucrose and solidified with 0.8% Gelrite agar (DUCHEFA). Seeds were kept in a growth chamber at 26 °C under a 16/8-h photoperiod at 120 µmol/m^2^s for 2 weeks until cotyledons appeared.

### Plasmid construction and generation of transgenic hairy roots

All PCR amplifications used for plasmid construction were performed with Recombinant Taq DNA polymerase (THERMO SCIENTIFIC, Paisley, UK) according to the manufacturer’s instructions. To amplify *LOX1.2*, *AOS1, OPR2* and* αDOX1* promoter regions, tomato genomic DNA was used as a template for PCR, with the following primers: *LOX1.2* FOR: 5′-TAT CAT ATA AGT GAG CTC GGA CTT ACT*-*3′, REV: 5′-AGC TGA CTG GCC CGG GTT TTC CTC AGA AAA AGT TTC-3′, with SacI and SmaI restriction sites; *AOS1* FOR: 5′-CGT TTT CAC AGG TCG AAT TCA ACG CCG T-3′, REV: 5′-AGG TAC CTA GCC CGG GTT CTA TTA GAA AAA AAT CAA-3′, with EcoRI and SmaI restriction sites; *OPR2* FOR: 5′-CTT TTA TGA ATG GTG GTA CCC TTT CCA-3′, REV: 5′-AGG TAC CTA GCC CGG GAC TTG ACA ACT AAA A-3′, with Kpn1 and SmaI restriction sites; *αDOX1* FOR:5′-TTG GGA GAG AGG AGC TCG ACA ATT TTT-3′, REV-5′-AGG TAC CTA GCC CGG GTG TTT ATA CGA-3′, with SacI and SmaI restriction sites; all for ≈ 1500 pb amplicons. *LOX1.2*, *AOS1, OPR2* and αDOX1 promoters were then cloned into the pUC19_Y vector^122^. The whole cassettes containing the specific gene promoters and the GUS reporter gene were then isolated by restriction digestion with SacI, EcoRI, KpnI and SacI (respectively) and SalI, to be cloned into the pCAMBIA2300 binary vector. The identity, orientation, and junctions of the resulting constructs p*LOX1.2*:GUS, p*AOS1:*GUS*, pOPR2:*GUS and *pαDOX:*GUS were confirmed by their digestion patterns. The pCAMBIA2300 empty-vector control and the four constructs were subsequently used for *Rhizobium rhizogenes*-mediated transformation^123^.

### *Rhizobium rhizogenes*-mediated root transformation

*R. rhizogenes* ATCC 15,834 strain was used for the transformation by heat-shock method^124^. Individual cotyledons were excised from 15- to 20-day-old tomato seedlings grown as described above and immersed in a 2-day-old *R. rhizogenes* suspension for incubation at 28 °C for 2 h, with agitation at 100 rpm. The excised cotyledons then were placed on standard-strength Gamborg’s B5 salt media for 3 days for co-cultivation, and then transferred to B5 agar media supplemented with the antibiotics kanamycin (50 mg/mL) (DUCHEFA, Haarlem, the Netherlands) and timentin (15:1) at 300 mg/mL (DUCHEFA, Haarlem, the Netherlands). After 7–10 days of incubation in the dark at 25 °C, roots emerged from the wounded surface of the cotyledons. Hairy roots were transferred to Gamborg’s B5 medium containing 0.8% Gelrite and kanamycin (50 mg/mL). For nematode-infection experiments, transformed roots were subcultured in antibiotic-free media for 2 weeks, and 300 freshly hatched sterile *M. javanica* juveniles were used to inoculate the transgenic root lines, and root samples were taken at the designated time points for GUS assessment.

### GUS bioassay

Two-week-old hairy root lines carrying the promoter GUS constructs were inoculated as described by Chinnapandi et al.^125^, and assayed histochemically for GUS activity at the designated times after infection with 300 sterile freshly hatched pre-parasitic *M. javanica* J2*.*

### Plant oxylipin and hormone extraction

Tomato (*Solanum lycopersicon* cv. Avigail 870) seeds were sterilized and planted as described. Emerging roots were cut into 2-cm long segments, subcultured on Gamborg’s B5 medium and kept in the dark for 2 weeks. Root systems were inoculated with freshly hatched *M. javanica* J2 and galls were collected 5, 15 and 28 DAI. The noninoculated roots were collected as controls. Five independent biological replicates with a total of 100–130 mg of gall samples from inoculated and noninoculated tomato roots at the selected time points were collected into a 1.5-mL tube and kept at − 80 °C until further analysis. Samples were weighed and all of the data were normalized to the relative weight of those frozen tissues.

Oxylipins/hormones were extracted from each sample in liquid N_2_ using the phytohormone-extraction protocol reported by Yang et al.^126^ with the following modifications: 500 µL of phytohormone extraction buffer (1-propanol:water:HCl at 2:1:0.002 v/v) containing 500 nM deuterated internal standards: d-ABA ([^2^H_6_]( +)-cis, trans-ABA; [OLCHEM]), d-IAA ([^2^H_5_] indole-3-acetic acid, OLCHEM), d-JA (2,4,4-d3; acetyl-2,2-d2 JA; CDN Isotopes), and d-SA (d6-SA, SIGMA) were added to 100–130 mg gall tissue^127^. The gall tissue was homogenized at 6000 rpm for 30 s, twice. Samples were agitated for 30 min at 4 °C in the dark and then 500 µL dichloromethane was added and samples were agitated again for 30 min at 4 °C in the dark. Samples were centrifuged at 13,000* g* for 5 min and the lower layer was collected into a glass vial for complete evaporation under a N_2_ gas stream. Samples were resuspended in 150 µL methanol, shaken for 1 min and then centrifuged in a 1.5-mL microcentrifuge tube at 14,000* g* for 2 min to pellet any debris. Supernatant (100 µL) was collected into an autosampler vial for injection into a SCIEX API 3200 LC–MS/MS with a C18 column for chromatography and electrospray ionization. Peaks were integrated using Analyst 1.6.2 software and metabolites were quantified against internal standards^41,127^. Identification of fatty acid peaks was verified by comparison of the mass spectra to authentic standards. Noninoculated tomato roots served as controls.

### Real-time qPCR analysis

For the qRT-PCR experiments, we removed contaminating genomic DNA from RNA with the Turbo DNA-Free Kit from AMBION (APPLIED BIOSYSTEMS). DNA-free RNA (1 μg) was converted to first-strand cDNA using the Verso cDNA Synthesis Kit (ABGENE, Epsom, UK), and reactions were performed using ABsolute SYBR Green ROX Mix (ABGENE). Primers for qRT-PCR experiments were designed with Primer Express software (APPLIED BIOSYSTEMS, Table 1S). A total volume of 10 μL contained 3.4 µL cDNA, consisting of 1 × SYBR-Green ROX Mix (ABGENE) and 150 nM forward primer and 150 nM reverse primer subjected to real-time PCR (Rotor-Gene RG-3000, CORBETT RESEARCH) using 0.1 mL 4-tube strips & caps (AXYGEN, Union City, CA, USA). All PCR cycles began with 2 min at 50 °C, then 10 min at 95 °C, followed by 40 cycles of 10 s at 95 °C and 1 min at 60 °C. After the PCR, a melting curve was generated by gradually increasing the temperature to 95 °C to test for amplicon specificity. For qRT-PCR, a mixture of all cDNAs was used for all treatments as a template for calibration curves designed for each pair of primers. Each reaction was performed in triplicate and the results represent the mean of two independent biological experiments. Two reference genes, *EF-1α* (GenBank accession no. U94493.1) and *18S *(GenBank accession no. AF442193.1), were used as endogenous controls for gene-expression analysis. Transcript levels were normalized for each sample with the geometric mean of the corresponding selected reference genes. All of the reference genes were confirmed to display minimal variation across the treatment and were the most stable reference genes from a set of tested genes in a given cDNA sample. Values were expressed as the increase or decrease in level relative to a calibration sample. A negative control PCR without cDNA template was also run to confirm the absence of nonspecific PCR products (NTC) No Template Control^127^. The same process was performed for qRT-PCR of the expression of the nematode effectors *TAG lipase* and *MLT-10*.

### FISH

Freshly hatched pre-parasitic *M. javanica* J2 were exposed to 9-HOT diluted in MES buffer to a final concentration of 10 mM, or to 0.01 M MES buffer, for 3 h; all samples were washed with 0.01 M MES buffer. The FISH procedure followed the method of Sakurai et al.^128^ with slight modifications^129^. The J2 were dissected manually with a razor blade and transferred to Carnoy's fixative (chloroform:ethanol:glacial acetic acid, 6:3:1, v/v) and fixed overnight. The samples were then decolorized in 6% (v/v) hydrogen peroxide in ethanol for 2 h and hybridized overnight in hybridization buffer (20 mM Tris–HCl pH 8.0, 0.9 M NaCl, 0.01% w/v SDS, 30% v/v formamide) containing 10 pmol fluorescent probe/mL. Based on the transcriptome sequences of interest, DNA probes were designed using Primer Express 3.0.1 software and checked for specificity using BLASTn (NCBI); TAG lipase (nematode effector) Cy5 (5′-Cy5-AATTGATGTTCGTGCAGACCAT-3′) and *MLT-10*-like Cy5 (5′-Cy5′-AGACAAAAGGGTGCAGAACGA-3′) were used as probes to target *M. javanica* J2. The stained samples were submerged in hybridization buffer supplemented with DAPI (0.1 mg/mL in 1X PBS) and transferred to a slide with liquid blocker, covered, sealed with nail polish and viewed under a confocal microscope.Detection specificity was confirmed using *M. javanica* exposed to 0.01 M MES buffer only as a control.

## Supplementary Information

Supplementary Figure 1.

Supplementary Figure 2.

Supplementary Figure 3.

Supplementary Figure 4.

Supplementary Table.
