# Supplementary material for: Oxylipins are implicated as communication signals in tomato–root-knot nematode (Meloidogyne javanica) interaction
Source: Sci Rep. 2021 Jan 11;11:326. doi: 10.1038/s41598-020-79432-6 (PMC7801703; doi:10.1038/s41598-020-79432-6)
Supplement: Supplementary file 1 — Supplementary Figure 1. [file 41598_2020_79432_MOESM1_ESM.pptx]

## Slide 1
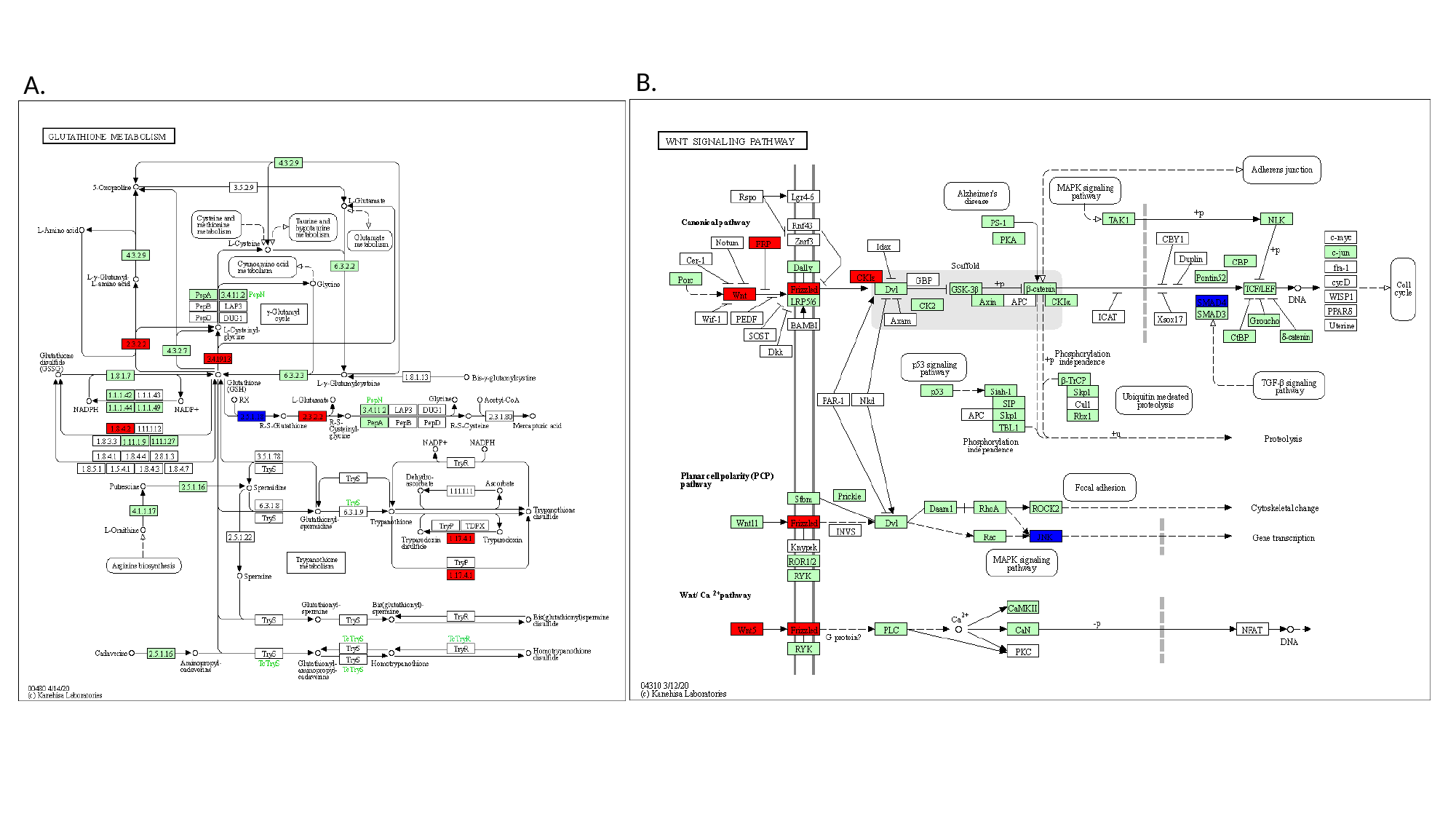

B.
A.

## Slide 2
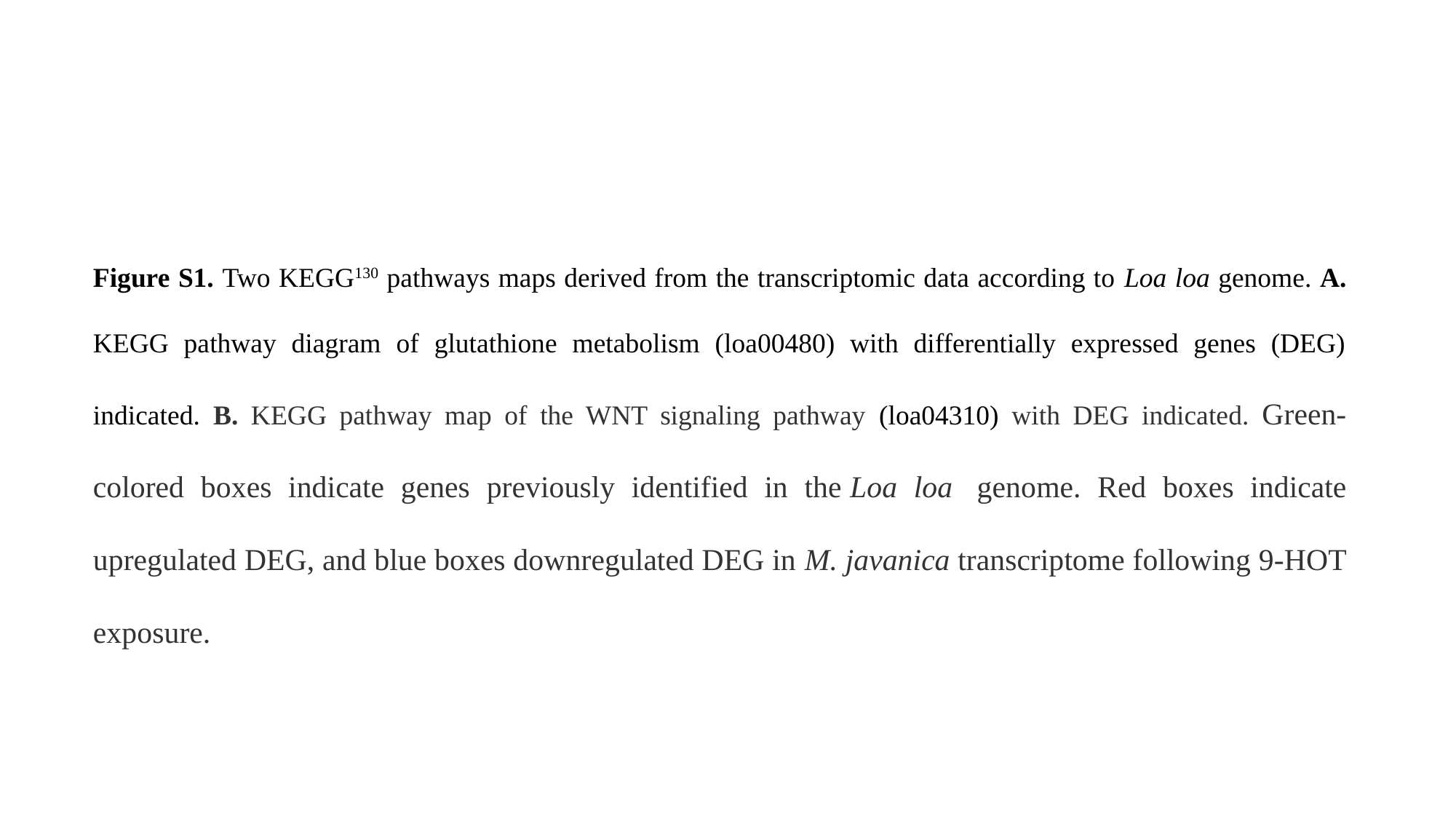

Figure S1. Two KEGG130 pathways maps derived from the transcriptomic data according to Loa loa genome. A. KEGG pathway diagram of glutathione metabolism (loa00480) with differentially expressed genes (DEG) indicated. B. KEGG pathway map of the WNT signaling pathway (loa04310) with DEG indicated. Green-colored boxes indicate genes previously identified in the Loa loa  genome. Red boxes indicate upregulated DEG, and blue boxes downregulated DEG in M. javanica transcriptome following 9-HOT exposure.
