# Supplementary material for: Oxylipins are implicated as communication signals in tomato–root-knot nematode (Meloidogyne javanica) interaction
Source: Sci Rep. 2021 Jan 11;11:326. doi: 10.1038/s41598-020-79432-6 (PMC7801703; doi:10.1038/s41598-020-79432-6)
Supplement: Supplementary file 2 — Supplementary Figure 2. [file 41598_2020_79432_MOESM2_ESM.pptx]

## Slide 1
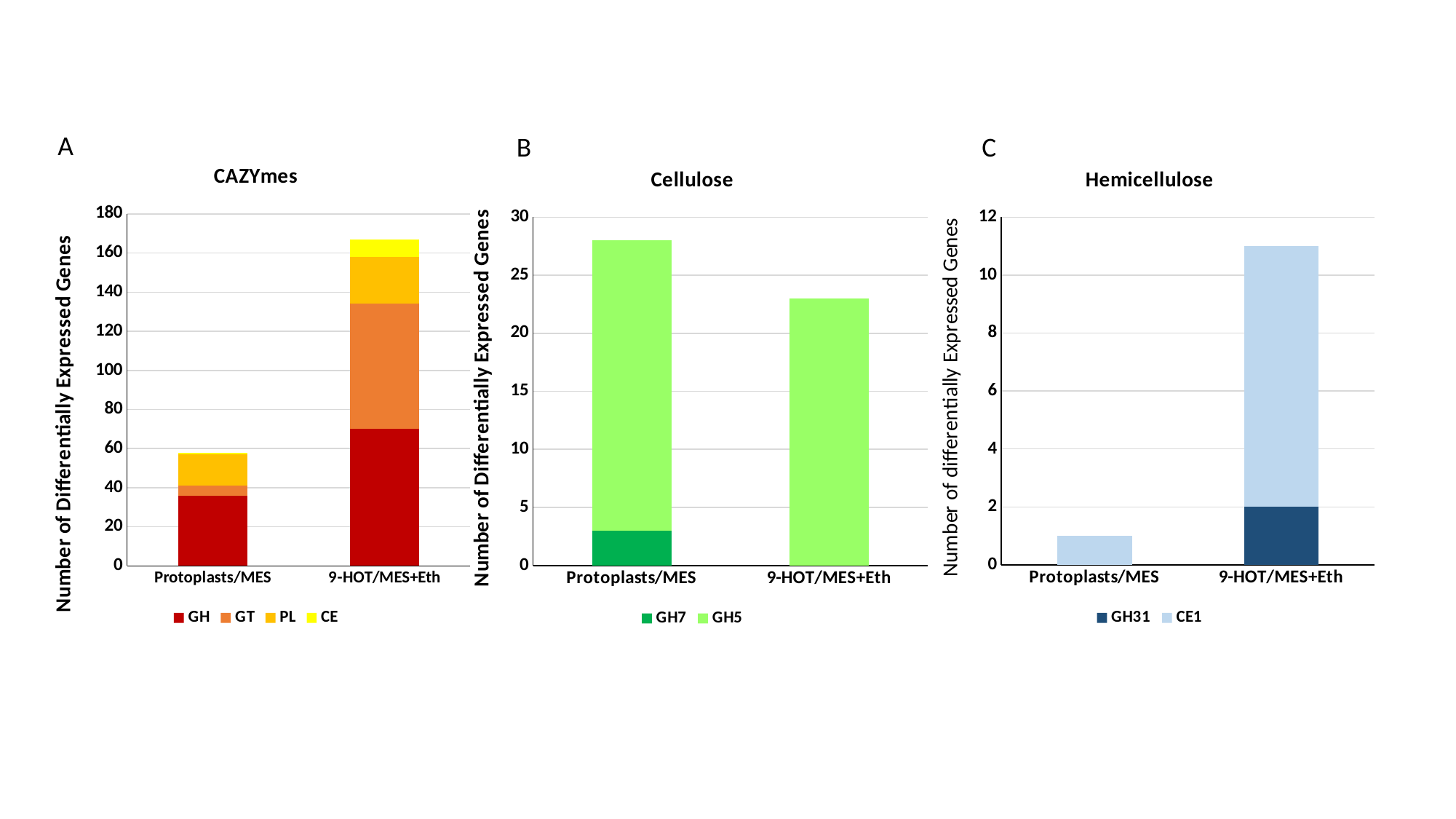

A
C
B
### Chart: Cellulose
| Category | GH74 | GH12 | GH7 | GH5 |
|---|---|---|---|---|
| Protoplasts/MES | 0.0 | 0.0 | 3.0 | 25.0 |
| 9-HOT/MES+Eth | 0.0 | 0.0 | 0.0 | 23.0 |
### Chart: Hemicellulose
| Category | GH31 | CE1 |
|---|---|---|
| Protoplasts/MES | 0.0 | 1.0 |
| 9-HOT/MES+Eth | 2.0 | 9.0 |
### Chart: CAZYmes
| Category | GH | GT | PL | CE | CBM | AA |
|---|---|---|---|---|---|---|
| Protoplasts/MES | 36.0 | 5.0 | 16.0 | 1.0 | 0.0 | 0.0 |
| 9-HOT/MES+Eth | 70.0 | 64.0 | 24.0 | 9.0 | 0.0 | 0.0 |

## Slide 2
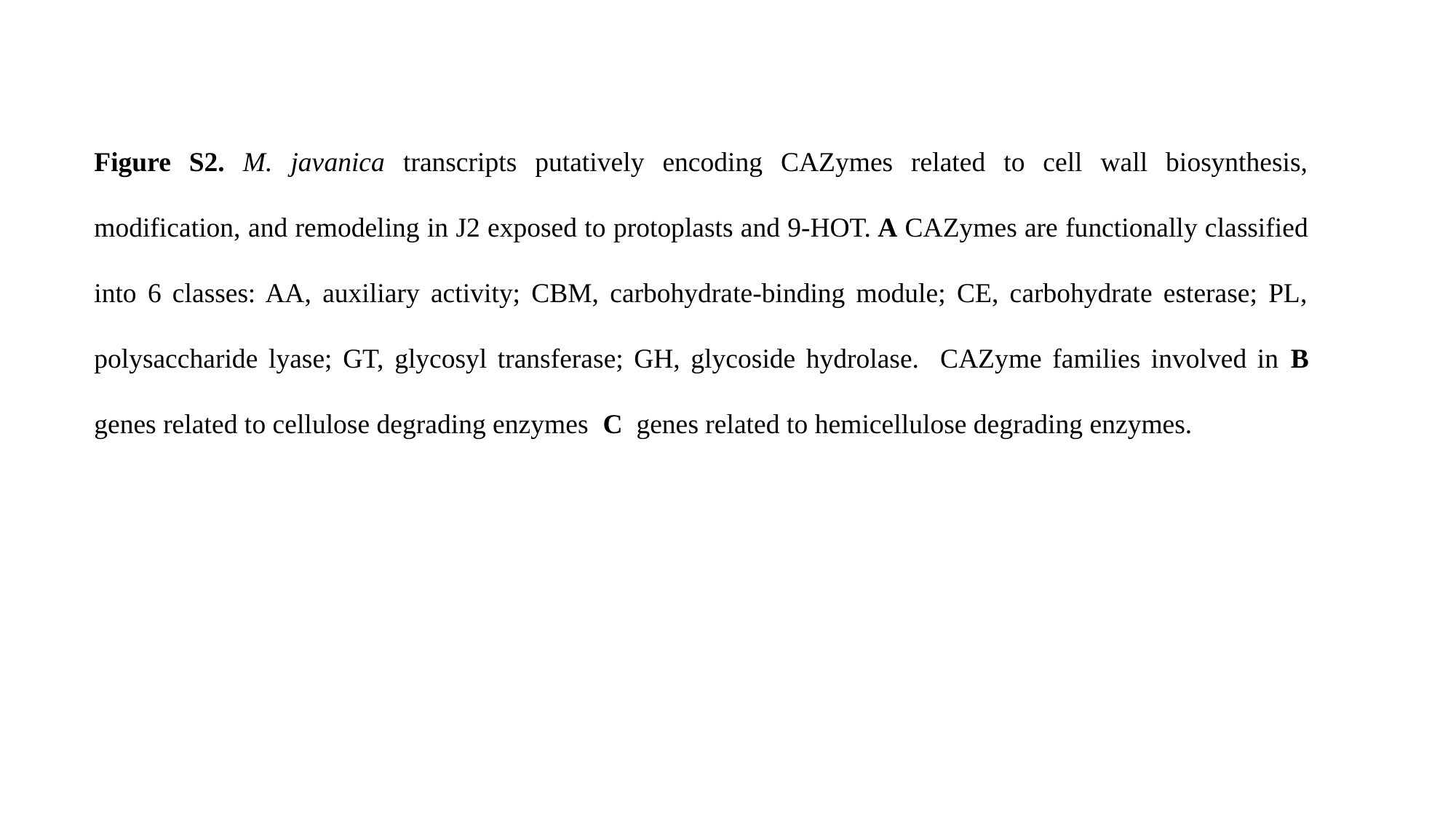

Figure S2. M. javanica transcripts putatively encoding CAZymes related to cell wall biosynthesis, modification, and remodeling in J2 exposed to protoplasts and 9-HOT. A CAZymes are functionally classified into 6 classes: AA, auxiliary activity; CBM, carbohydrate-binding module; CE, carbohydrate esterase; PL, polysaccharide lyase; GT, glycosyl transferase; GH, glycoside hydrolase. CAZyme families involved in B genes related to cellulose degrading enzymes C genes related to hemicellulose degrading enzymes.
