# Supplementary material for: Oxylipins are implicated as communication signals in tomato–root-knot nematode (Meloidogyne javanica) interaction
Source: Sci Rep. 2021 Jan 11;11:326. doi: 10.1038/s41598-020-79432-6 (PMC7801703; doi:10.1038/s41598-020-79432-6)
Supplement: Supplementary file 3 — Supplementary Figure 3. [file 41598_2020_79432_MOESM3_ESM.pptx]

## Slide 1
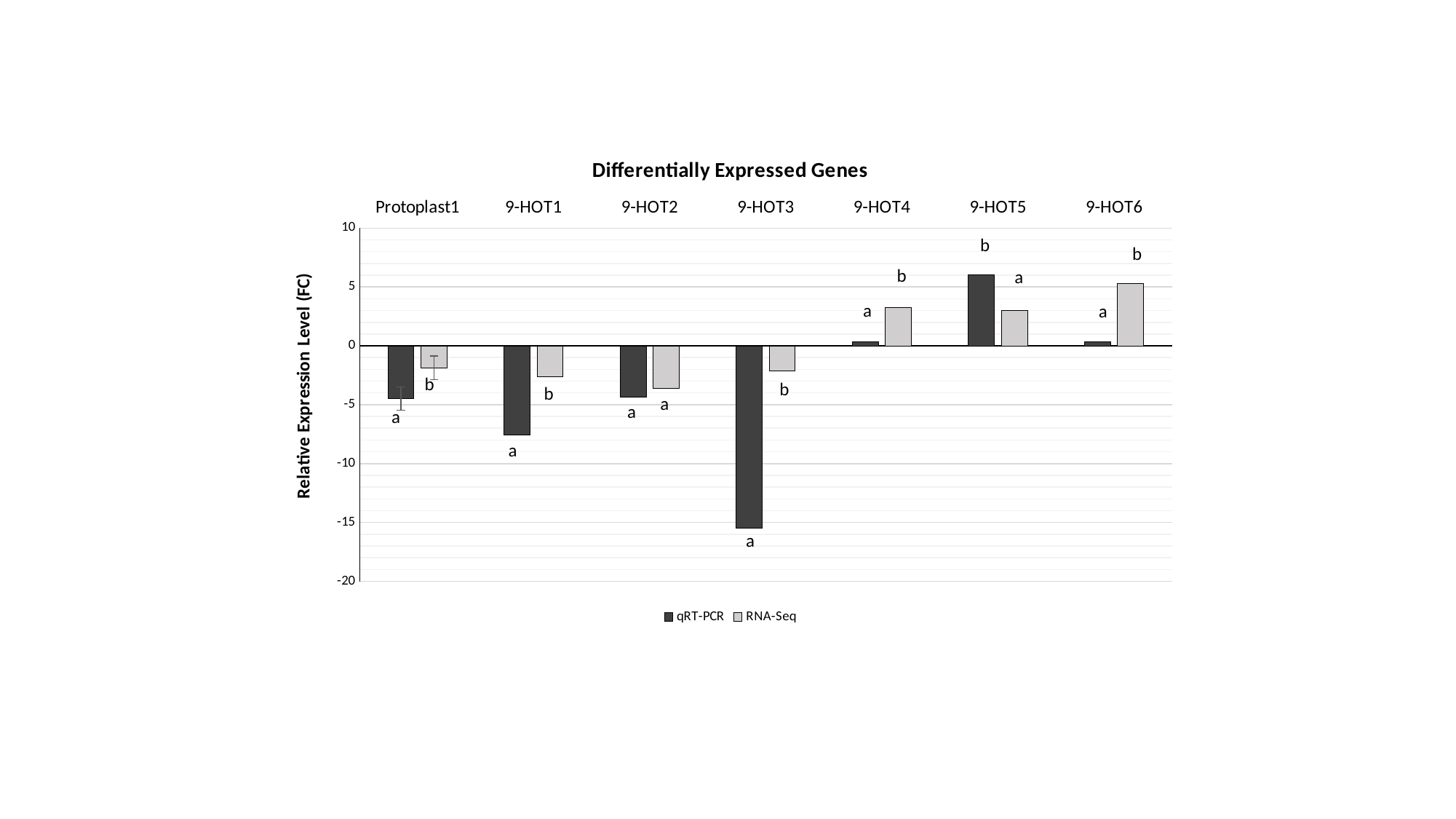

### Chart: Differentially Expressed Genes
| Category | qRT-PCR | RNA-Seq |
|---|---|---|
| Protoplast1 | -4.479853162632141 | -1.8666809007283403 |
| 9-HOT1 | -7.603729823558631 | -2.6234583235210636 |
| 9-HOT2 | -4.331017603752519 | -3.619885337650483 |
| 9-HOT3 | -15.464098593024978 | -2.1459820633134123 |
| 9-HOT4 | 0.32169451481465006 | 3.242852169324529 |
| 9-HOT5 | 6.023305787122208 | 3.0111466785250487 |
| 9-HOT6 | 0.3386612988734462 | 5.259738286622885 |b
b
b
a
a
a
b
b
b
a
a
a
a
a

## Slide 2
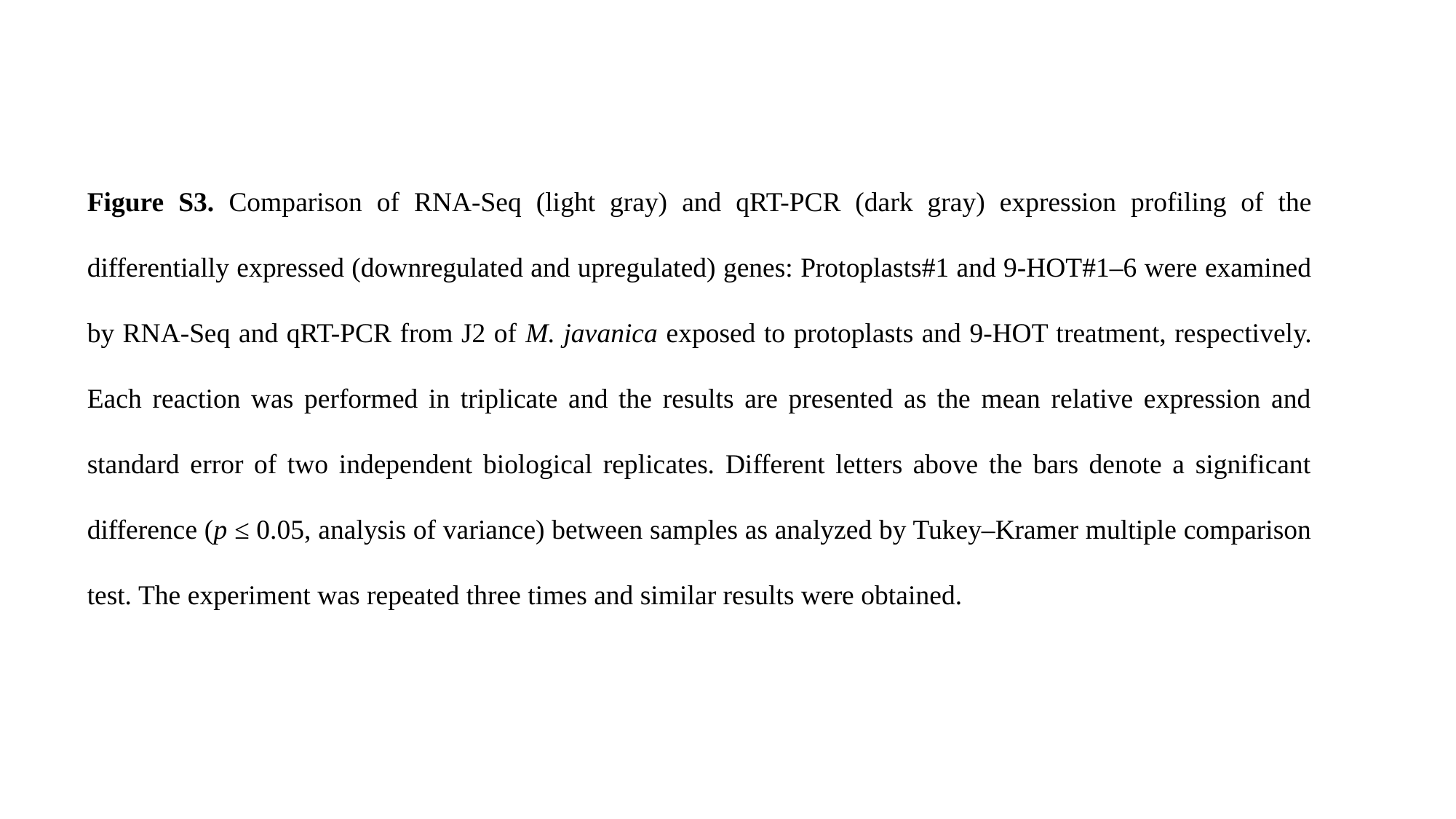

Figure S3. Comparison of RNA-Seq (light gray) and qRT-PCR (dark gray) expression profiling of the differentially expressed (downregulated and upregulated) genes: Protoplasts#1 and 9-HOT#1–6 were examined by RNA-Seq and qRT-PCR from J2 of M. javanica exposed to protoplasts and 9-HOT treatment, respectively. Each reaction was performed in triplicate and the results are presented as the mean relative expression and standard error of two independent biological replicates. Different letters above the bars denote a significant difference (p ≤ 0.05, analysis of variance) between samples as analyzed by Tukey–Kramer multiple comparison test. The experiment was repeated three times and similar results were obtained.
