# Supplementary material for: Oxylipins are implicated as communication signals in tomato–root-knot nematode (Meloidogyne javanica) interaction
Source: Sci Rep. 2021 Jan 11;11:326. doi: 10.1038/s41598-020-79432-6 (PMC7801703; doi:10.1038/s41598-020-79432-6)
Supplement: Supplementary file 4 — Supplementary Figure 4. [file 41598_2020_79432_MOESM4_ESM.pptx]

## Slide 1
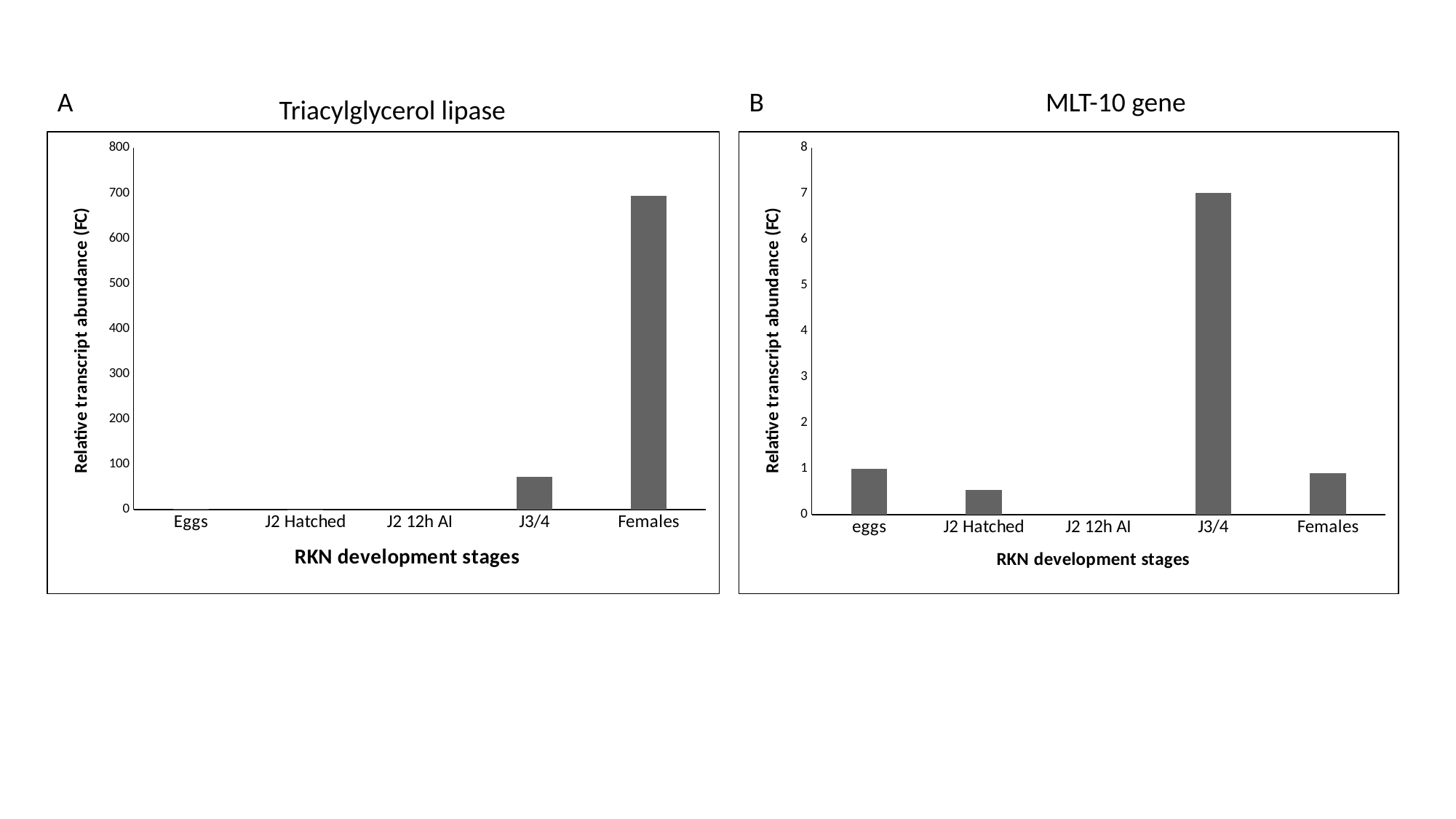

A
B
MLT-10 gene
Triacylglycerol lipase
### Chart
| Category | Relative transcript abundance (FC) |
|---|---|
| Eggs | 1.0 |
| J2 Hatched | 0.04696441521167206 |
| J2 12h AI | 0.0 |
| J3/4 | 73.05765906804756 |
| Females | 694.0477611464518 |
### Chart
| Category | Relative transcript abundance (FC) |
|---|---|
| eggs | 1.0 |
| J2 Hatched | 0.544941 |
| J2 12h AI | 0.0 |
| J3/4 | 7.02548 |
| Females | 0.901356 |

## Slide 2
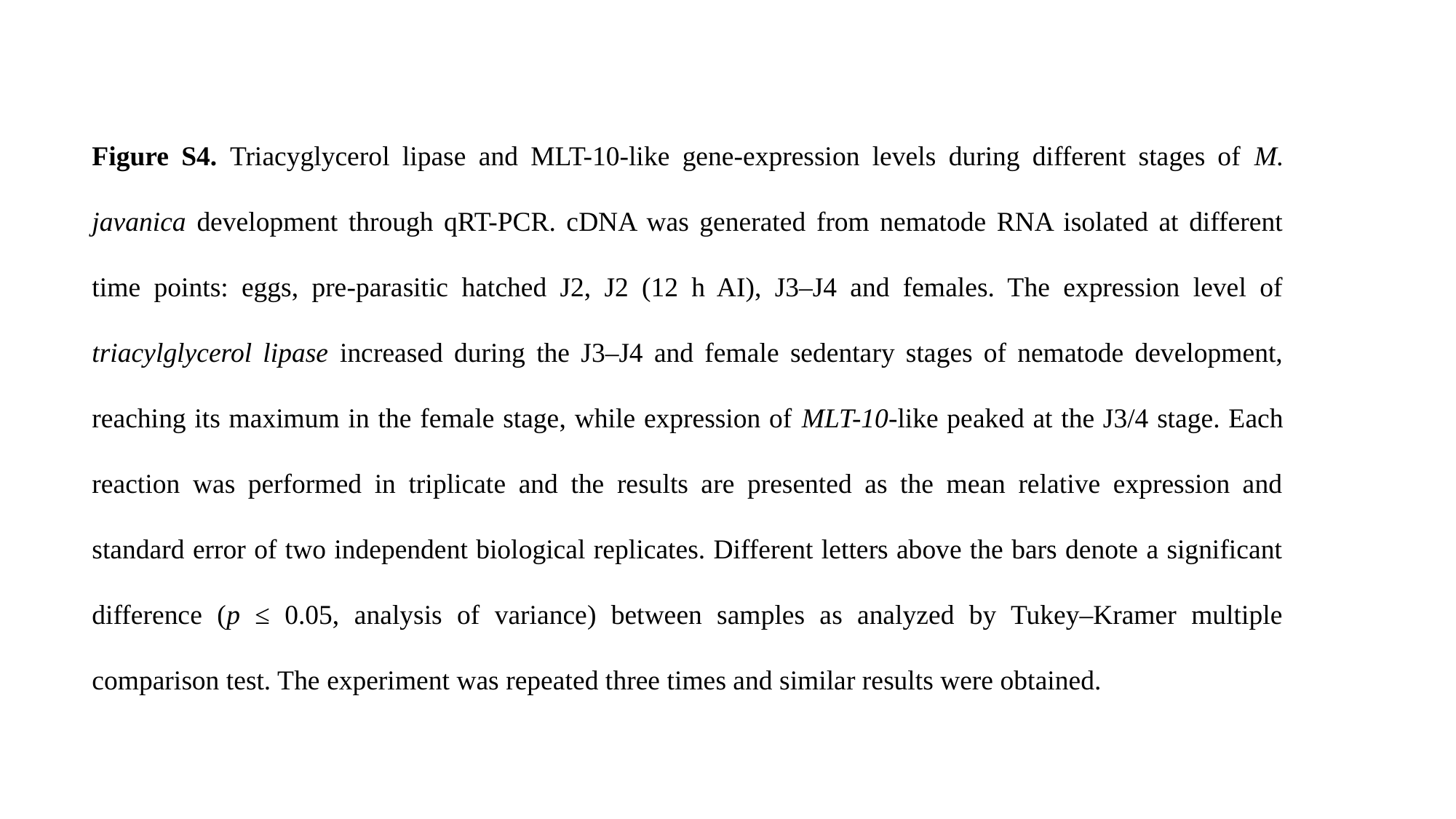

Figure S4. Triacyglycerol lipase and MLT-10-like gene-expression levels during different stages of M. javanica development through qRT-PCR. cDNA was generated from nematode RNA isolated at different time points: eggs, pre-parasitic hatched J2, J2 (12 h AI), J3–J4 and females. The expression level of triacylglycerol lipase increased during the J3–J4 and female sedentary stages of nematode development, reaching its maximum in the female stage, while expression of MLT-10-like peaked at the J3/4 stage. Each reaction was performed in triplicate and the results are presented as the mean relative expression and standard error of two independent biological replicates. Different letters above the bars denote a significant difference (p ≤ 0.05, analysis of variance) between samples as analyzed by Tukey–Kramer multiple comparison test. The experiment was repeated three times and similar results were obtained.
