# Supplementary material for: Oxylipins are implicated as communication signals in tomato–root-knot nematode (Meloidogyne javanica) interaction
Source: Sci Rep. 2021 Jan 11;11:326. doi: 10.1038/s41598-020-79432-6 (PMC7801703; doi:10.1038/s41598-020-79432-6)
Supplement: Supplementary file 5 — Supplementary Table. [file 41598_2020_79432_MOESM5_ESM.docx]

**Table 1S**. List of all qRT-PCR forward and reverse primers used in this study and the housekeeping genes that served as reference genes for validation of selected DEGs. Protoplast#1 and 9-HOT#1-3 were down-regulated genes and 9-HOT#4-7 were up-regulated genes.
